# Molecular imaging of the kinetics of hyperactivated ERK1/2-mediated autophagy during acquirement of chemoresistance

**DOI:** 10.1038/s41419-021-03451-y

**Published:** 2021-02-08

**Authors:** Aniketh Bishnu, Pratham Phadte, Ajit Dhadve, Asmita Sakpal, Bharat Rekhi, Pritha Ray

**Affiliations:** 1 Imaging Cell Signalling & Therapeutics Lab, Advanced Centre for Treatment, Research and Education in Cancer, TMC, Navi Mumbai, 410210 India; 2 Homi Bhabha National Institute, Anushakti Nagar, Mumbai, 400094 India; 3 Department of Pathology, Tata Memorial Hospital, Mumbai, 400012 India

**Keywords:** Ovarian cancer, Macroautophagy

## Abstract

Alterations in key kinases and signaling pathways can fine-tune autophagic flux to promote the development of chemoresistance. Despite empirical evidences of strong association between enhanced autophagic flux with acquired chemoresistance, it is still not understood whether an ongoing autophagic flux is required for both initiation, as well as maintenance of chemoresistance, or is sufficient for one of the either steps. Utilizing indigenously developed cisplatin–paclitaxel-resistant models of ovarian cancer cells, we report an intriguing oscillation in chemotherapy-induced autophagic flux across stages of resistance, which was found to be specifically elevated at the early stages or onset of chemoresistance. Conversely, the sensitive cells and cells at late stages of resistance showed stalled and reduced autophagic flux. This increased flux at early stages of resistance was found to be dictated by a hyperactive ERK1/2 signaling, which when inhibited either pharmacologically (U0126/Trametinib) or genetically, reduced p62 degradation, number of LC3^+ve^LAMP1^+ve^ puncta, autophagolysosome formation, and led to chemo-sensitization and apoptosis. Inhibition of ERK1/2 activation also altered the level of UVRAG and Rab7, the two key proteins involved in autophagosome–lysosome fusion. Noninvasive imaging of autophagic flux using a novel autophagy sensor (mtFL-p62 fusion reporter) showed that combinatorial treatment of platinum–taxol along with Trametinib/chloroquine blocked autophagic flux in live cells and tumor xenografts. Interestingly, Trametinib was found to be equally effective in blocking autophagic flux as chloroquine both in live cells and tumor xenografts. Combinatorial treatment of Trametinib and platinum–taxol significantly reduced tumor growth. This is probably the first report of real-time monitoring of chemotherapy-induced autophagy kinetics through noninvasive bioluminescence imaging in preclinical mouse model. Altogether our data suggest that an activated ERK1/2 supports proper completion of autophagic flux at the onset of chemoresistance to endure initial chemotherapeutic insult and foster the development of a highly chemoresistant phenotype, where autophagy becomes dispensable.

## Introduction

Macroautophagy, herein referred as autophagy, a stress-induced cellular catabolic process is reported to promote acquirement of chemoresistance in various malignancies, including epithelial ovarian cancer (EOC)^1,2^. Chemoresistance, a multistep dynamic process, involves activation of several key kinase pathways like AMPK, mTOR1, AKT, and ERK1/2, which in turn can modulate and fine-tune the autophagic flux in cancer cells^3–5^. In contrast to AKT, which promotes development of chemoresistance in several cancers but negatively regulates autophagy, the MAPK/ERK1/2 signaling plays a contextual role in modulating autophagic flux^6–9^. ERK1/2 promotes autophagic flux in chemoresistant gastric, breast, and ovarian cancer cells, but downregulates the same in basal or BRAF inhibitor resistant lung, breast, and PDAC cells^10–12^. Unfortunately, all these studies reported a onetime relation between chemoresistance and autophagic flux, and did not investigate the dynamic association of autophagy across different stages of resistance. Thus understanding the role of autophagy during the development of chemoresistance and its regulation through associated kinase/s would enable us to design and implement optimal therapeutic regimen at precise therapeutic window to combat chemoresistance.

Autophagy is critical in maintaining cellular homeostasis and increases significantly as a survival response to various stresses. The dynamicity of autophagosome formation and autophagosome–lysosome fusion is routinely captured in real time through various florescent-based reporters (GFP-LC3, mCherry-GFP-LC3, and GFP-LC3-RFP-LC3ΔG) by live cell imaging^13–15^. However, cell line-based studies cannot recapitulate the organ biology of a live organism and these fluorescent-based sensors experience severe limitations due to auto-florescence, tissue absorption, and signal attenuation for noninvasive in vivo imaging. Luciferase-based reporters can side step these limitations and efficiently be used for real-time imaging of autophagic flux in preclinical mouse models^16,17^. Among the currently available luciferase-based autophagy sensors (LC3-Rluc^C124A^, p62-luc2p, and poly80/poly19-FL2) only poly80/poly19-FL2 sensor was validated for ratio-metrically monitoring autophagy in vivo; however, the autophagy-independent degradation of FL2 and poly-glutamine repeats may lead to inaccurate measurement of autophagic flux^18–20^. Thus, there is a pressing need to develop an in vivo autophagy sensor, which will reliably portray the progression/stalling of autophagic flux noninvasively in preclinical animal models.

In this report, we identified differential levels of autophagic flux at different stages of cisplatin–paclitaxel resistanct A2780 and OAW42 EOC cellular models. Utilizing these indigenously developed chemoresistant models, we previously showed that an upregulated IGF1R expression was beneficial for the cancer cells to survive therapeutic stress at the onset of resistance (early resistant or Dual^ER^ cells), but was dispensable when they attained a highly resistant phenotype (late resistant or Dual^LR^ cells) with high levels of activated AKT^21^. Herein, we show that both sensitive and Dual^LR^ cells exhibited stalled or reduced autophagy after drug treatment, but the Dual^ER^ cells showed sustained autophagic flux, governed by hyperactive ERK1/2 kinase through appropriate autophagosome–lysosome fusion via regulation of UV radiation resistance associated (UVRAG) and Rab7. Utilizing a novel autophagy sensor comprised of a mutant thermostable firefly luciferase fused with p62 (mtFL-p62), we further showed that combinatorial treatment of cisplatin-paclitaxel (CisPac) with either Trametinib, a clinically approved MEK1/2 inhibitor or chloroquine (CQ) led to p62 accumulation, while only chemotherapeutic drug treatment led to p62 degradation as monitored longitudinally by bioluminescence imaging in live cells and tumor-bearing mice. Further combinatorial treatment of CisPac with Trametinib significantly reduced tumor growth in comparison to single agent-treated and -untreated groups. To the best of our knowledge, this is the first report of noninvasive monitoring of chemotherapy-induced autophagic flux promoted by active ERK1/2 kinase from cells to preclinical mouse model, which might lay the future ERK1/2-targeted therapeutic strategies against chemoresistant EOC.

## Results

### Autophagic flux remains upregulated at the early stage of platinum–taxol resistance

**Fig. 1:**
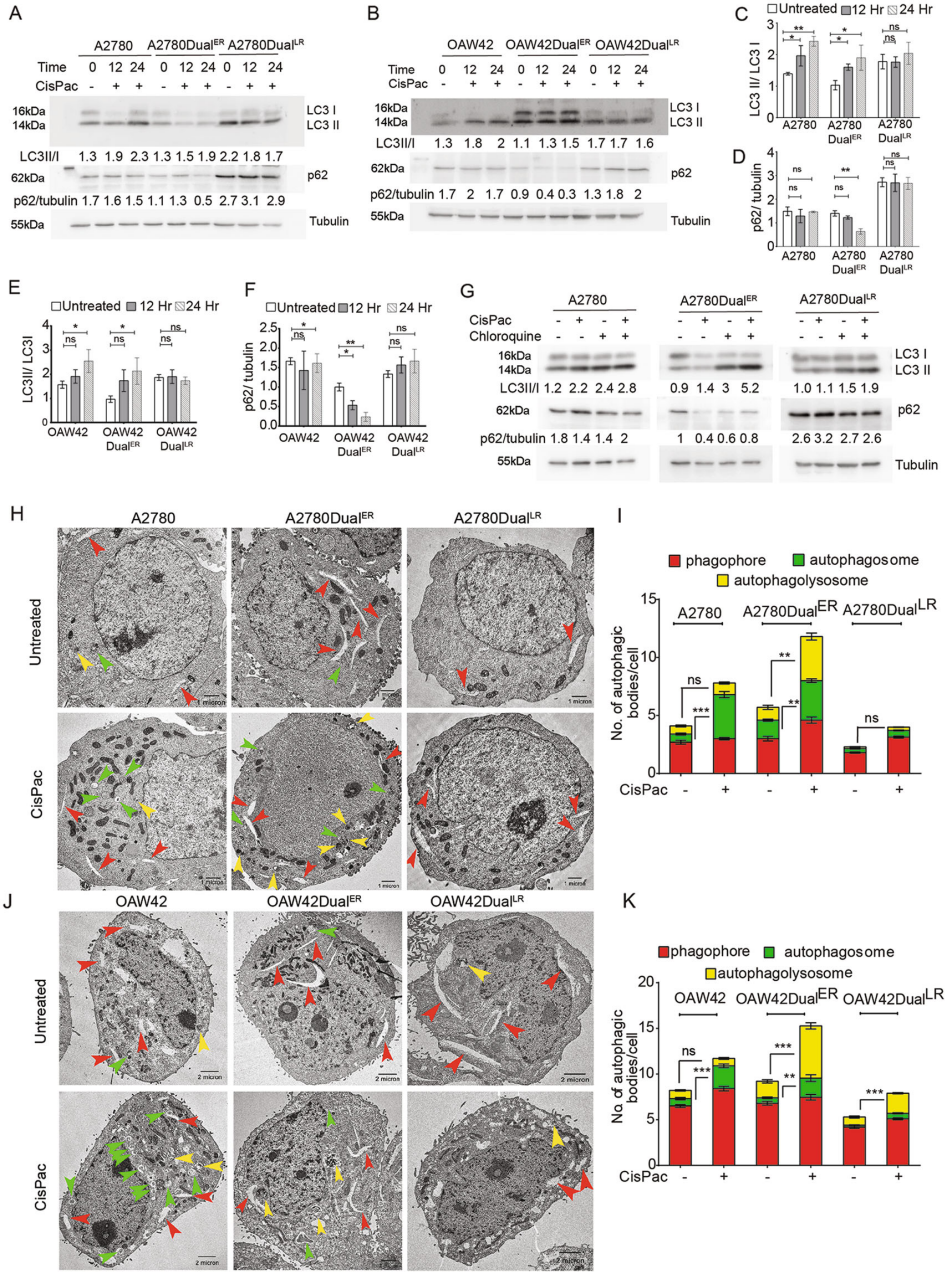
Autophagic flux remains upregulated in the early stage of resistance. **A**, **B** Immunoblot depicting increased LC3I–II conversion post CisPac treatment in both sensitive and early resistant cells of A2780 and OAW42 model. A2780Dual^LR^ and OAW42Dual^LR^ cells showed least change in LC3I–II conversion compared to untreated cells. p62 level reduced significantly in A2780Dual^ER^ and OAW42Dual^ER^ cells post 12 and 24 h of CisPac treatment. **C**–**F** Quantification of enhanced LC3II/LC3I ratio in A2780 [1.39 ± 0.04 (0 h) to 1.96 ± 0.15 (12 h) to 2.42 ± 0.08 (24 h)] and Dual^ER^ [1.03 ± 0.12 (0 h) to 1.6 ± 0.05 (12 h) to 1.9 ± 0.19 (24 h)] post CisPac treatment. Nonsignificant change in LC3II/LC3I ratio was observed in A2780Dual^LR^ cells. A similar trend in LC3II/LC3I ratio was observed in OAW42 chemoresistant model. Significant reduction in p62/tubulin ratio was specifically observed in A2780Dual^ER^ [1.39 ± 0.09 (0 h) to 0.63 ± 0.06 (24 h)] and OAW42Dual^ER^ cells [1 ± 0.08 (0 h) to 0.23 ± 0.05 (24 h)]. Nonsignificant changes in p62/tubulin ratio were observed in sensitive and Dual^LR^ cells. **G** Immunoblot depicting LC3I–II conversion and p62 level post 24 h of CQ treatment either alone or in combination with CisPac. Highest differences in LC3I–II conversion and p62 accumulation were observed in A2780Dual^ER^ cells treated with CisPac in presence and absence of CQ compared to sensitive and A2780Dual^LR^ cells. **H**, **J** Representative electron microscopy images of sensitive, early and late resistant A2780 and OAW42 cells with or without CisPac treatment depicting individual autophagic structure phagophore (red arrow), autophagosome (green arrow), and autophagolysosome (yellow arrow). **I**, **K** Graph depicting significantly higher autophagic bodies in Dual^ER^ cells (12 ± 1.39/cell and 15 ± 1.11/cell) compared to sensitive (8 ± 1.10/cell and 12 ± 1.02) and Dual^LR^ cells (3 ± 0.59/cell and 8 ± 0.58/cells) of A2780 and OAW42 models, respectively. Increased formation of autophagosomes was observed in A2780 (5.3-fold) and OAW42 (3.1-fold) cells post CisPac treatment, while increased numbers of both autophagosomes (2.1-fold and 3.4-fold) and autophagolysosomes (3.4-fold and 3.2-fold) were found in A2780Dual^ER^ and OAW42Dual^ER^ cells, respectively. A2780Dual^LR^ and OAW42Dual^LR^ cells showed minimal increase in both autophagosomes and autophagolysosomes (*n* = 10 cells/group, data represent mean ± SEM of at least two independent experiments, ns indicates nonsignificant, *p < 0.05, **p < 0.005, ***p < 0.0005 as calculated by unpaired *t* test).

In order to monitor the autophagic flux during the evolution of chemoresistance, we utilized indigenously built platinum–taxol-resistant A2780 and OAW42 cellular models which were developed by treating cells with successive and incremental doses of drugs over 6 months. These chemoresistant cells were then classified into sensitive, early (A2780/OAW42Dual^ER^) and late (A2780/OAW42Dual^LR^) resistant cells depending on their resistant indices^21^. When each stage of these models were treated with CisPac, a significantly increased LC3I–II conversion was observed in sensitive and Dual^ER^ cells, but minimally in Dual^LR^ cells at 12 and 24 h compared to untreated cells of both models (Fig. 1A–C, E). p62 degradation, an indicator of completion of autophagy, was most prominent in CisPac-treated Dual^ER^ cells of both models when quantified by the reduction in p62/tubulin ratio (Fig. 1A, B, D, F). CQ along with CisPac induced highest change in LC3 conversion and p62 stabilization in A2780Dual^ER^ cells compared to A2780 and A2780Dual^LR^ cells (Fig. 1G). Transmission electron microscopy (TEM) revealed significantly enhanced number of autophagic bodies in Dual^ER^ cells compared to sensitive and Dual^LR^ cells of A2780 and OAW42 models (Fig. 1H–K). CisPac treatment increased autophagosome number without any alteration in autophagolysosome number in A2780 and OAW42 cells, while a minimal increase in autophagosomes and autophagolysosomes was observed in A2780Dual^LR^ and OAW42Dual^LR^ cells. Interestingly, a distinct surge in numbers of both autophagosomes and autophagolysosomes were found in A2780Dual^ER^ and OAW42Dual^ER^ cells (Fig. 1I, K). Collectively, these data suggest the presence of chemotherapy-induced active autophagic flux exclusively at the onset of resistance.

### Activated ERK1/2 augments autophagic flux at early stages of platinum–taxol resistance

**Fig. 2:**
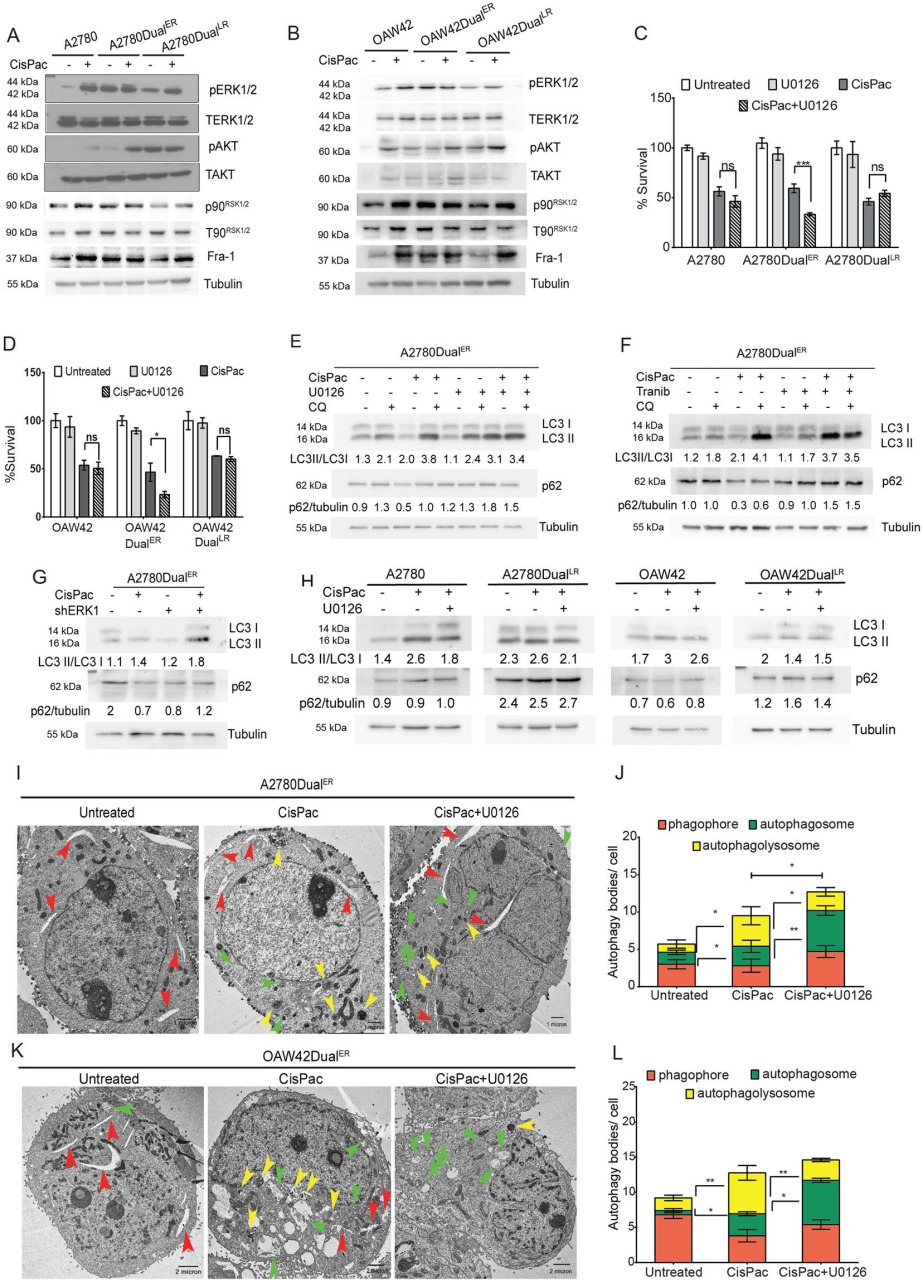
Hyperactivation of ERK1/2 sustains proper autophagic flux in early resistant cells. **A**, **B** Immunoblot analysis showed maximal basal level of phospho-ERK1/2, p90^RSK1/2^, and FRA-1 in A2780Dual^ER^ and OAW42Dual^ER^ cells compared to sensitive and late resistant cells of both A2780 and OAW42 model, while the basal level of AKT phosphorylation was highest in A2780Dual^LR^ and OAW42Dual^LR^ cells. **C**, **D** Cell survival assay signifying an additive cytotoxic effect of CisPac + U0126 over CisPac (IC50 dosage) and U0126 alone in A2780Dual^ER^ (26.15%) and OAW42Dual^ER^ (23.28%) cells, **E**, **F** Immunoblot depicting increased LC3 conversion and p62 accumulation in CisPac + CQ-treated A2780Dual^ER^ cells in comparison to cells treated with only CisPac, while application of CQ along with CisPac + U0126 or CisPac + Trametinib (Tranib) did not alter LC3 conversion or p62 level in comparison to cells treated with CisPac + U0126 or CisPac + Trametinib. **G** Increased LC3I–II conversion and p62 accumulation was observed in A2780Dual^ER/ERK1^ cells compared to the parental cells post 24 h of CisPac treatment. **H** Immunoblot depicting LC3I–II conversion and p62 accumulation in sensitive and late resistant A2780 and OAW42 cells treated with platinum–taxol alone or in combination of U0126. **I**, **K** Representative electron microscopy images of A2780Dual^ER^ and OAW42Dual^ER^ cells treated with CisPac alone or in combination of U0126 depicting individual autophagic structures phagophore (red arrow), autophagosome (green arrow), and autophagolysosome (yellow arrow), **J**, **L** Graphical representation of total and individual number of autophagic structures revealed increased number of phagophores (1.67- and 1.44-fold) and autophagosomes (2.1- and 2-fold) with a concomitant reduction (0.6- and 0.5-fold) in autophagolysosomes in A2780Dual^ER^ and OAW42Dual^ER^ cells post combinatorial treatment of CisPac and U0126 in comparison to only CisPac (*n* = 10 cells/group, data represent mean ± SEM of at least two independent experiments, ns indicates nonsignificant, *p < 0.05, **p < 0.005, ***p < 0.0005 as calculated by unpaired *t* test).

Exclusive presence of drug-induced autophagic flux at the onset of resistance prompted us to investigate the underlying molecular factors. Since we previously demonstrated an active IGF1R signaling in these cells, the activation status of two downstream signaling (MAPK/ERK and PI3K/AKT) were evaluated at different stages. Basal ERK1/2 activation was highest in A2780Dual^ER^ and OAW42Dual^ER^ cells which did not enhance after drug treatment. However, chemotherapy-induced ERK1/2 activation in sensitive and Dual^LR^ cells, despite having lower autophagic flux. Further increased basal levels of activated p90^RSK1/2^ and Fra-1, the two downstream targets of ERK1/2, were observed specifically in Dual^ER^ cells of both A2780 and OAW42 model, indicating presence of an activated ERK1/2 signaling in the onset of resistance (Fig. 2A, B). An additive toxicity was observed specifically in A2780Dual^ER^ and OAW42Dual^ER^ cells on combinatorial treatments (CisPac with ERK inhibitor-U0126) compared to CisPac and U0126 alone (Fig. 2C, D). Combinatorial treatment of U0126 and CisPac resulted in higher LC3I–II conversion and p62 accumulation compared to only CisPac-treated Dual^ER^ cells of both models (Fig. S1A, B). Addition of CQ along with CisPac and U0126 did not lead to further increase in LC3 conversion or p62 accumulation in comparison to CisPac + U0126, while combination of CQ along with CisPac increased LC3 conversion and p62 level compared to only CisPac, indicating a blockade in late stage of autophagy upon ERK1/2 inhibition (Fig. 2E). Similar results were observed when Trametinib, another ERK1/2 inhibitor, was used in the same conditions (Fig. 2F). Genetic knockdown of ERK1 reduced phosphorylated and total level of ERK1/2, and its downstream targets phospho p90^RSK^ and Fra-1 (Fig. S2). ERK1 knockdown (A2780Dual^ER/ERK1-KD^) increased LC3II and p62 accumulation compared to parental A2780Dual^ER^ cells post CisPac treatment (Fig. 2G). Combinatorial treatment of U0126 and CisPac in sensitive and Dual^LR^ cells did not show any significant changes in LC3I–II conversion or p62 level than their drug-treated counterparts in both the models, suggesting the role of basal ERK1/2 activation in completion of autophagy (Fig. 2H). Increased phagophores and autophagosomes with a concomitant reduction in autophagolysosomes were observed in A2780Dual^ER^ and OAW42Dual^ER^ cells post combinatorial treatment (CisPac + U0126) than platinum–taxol alone (Fig. 2I–L). Dual^LR^ cells showed reduced autophagic flux and highest AKT activation (Fig. 2A, B). Combinatorial treatment of AKT inhibitor with drugs induced higher LC3I–II conversion and p62 degradation in A2780Dual^LR^ and OAW42Dual^LR^ cells (Fig. S3A, B).

### Activated ERK1/2 kinase regulates autophagosome–lysosome fusion

**Fig. 3:**
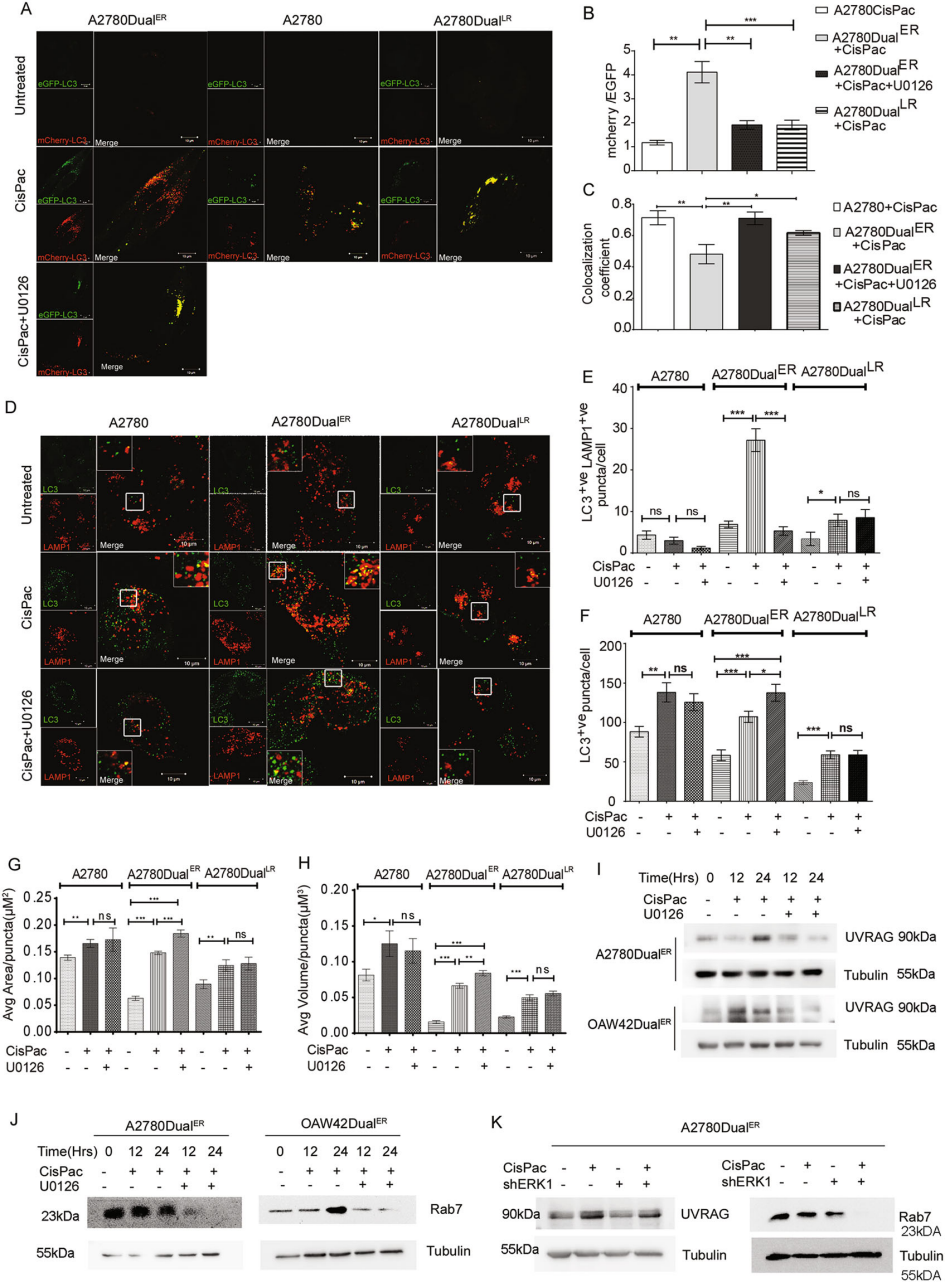
ERK1/2 activation regulates autophagosome–lysosome fusion during early stage of resistance. **A** Representative images of A2780, A2780Dual^ER^, and A2780Dual^LR^ cells expressing mCherry-EGFP-LC3 post 24 h of CisPac treatment alone or in combination with U0126. Yellow puncta [colocalization between mCherry (red) and EGFP (green)] indicate autophagosomes and red puncta (mCherry) denote autophagolysosome. A2780Dual^ER^ cells showed increased red puncta post CisPac treatment, while CisPac + U0126 treatment reduced number of red puncta and increased yellow puncta. Compared to the number of red puncta, higher numbers of yellow puncta were observed in A2780 and A2780Dual^LR^ cells. **B**, **C** Graphical representation of mCherry/EGFP ratio and Mander’s colocalization coefficient of mCherry and EGFP puncta. A2780Dual^ER^ cells showed increased mcherry/EGFP ratio (4.11) and reduced yellow puncta (colocalization coefficient: 0.48 + 0.06) post CisPac treatment, while CisPac + U0126 treatment reduced mcherry/EGFP ratio (1.91) and increased yellow puncta (colocalization coefficient: 0.70 ± 0.04). A2780 and A2780Dual^LR^ cells showed reduced mcherry/EGFP ratio (1.91 and 1.17, respectively) and increased yellow puncta (colocalization coefficient: 0.71 ± 0.04 and 0.61 ± 0.01, respectively; *n* = 35 cells/group). **D** Representative images of sensitive, early and late resistant cells of A2780 chemoresistant models, immunostained with LC3 (green, autophagosome) and LAMP1 (red, lysosome) post 24 h of CisPac treatment alone or in combination with U0126. Yellow puncta depict autophagosome–lysosome fusion. The white square insets represent the magnified images. **E** Graphical representation of the number of LC3^+ve^LAMP1^+ve^ puncta in sensitive, early and late resistant A2780 cells treated with CisPac alone or in combination U0126 for 24 h. CisPac treatment for 24 h induced maximal LC3^+ve^LAMP1^+ve^ puncta in A2780Dual^ER^ (27.16 ± 2.71) cells compared to sensitive (2.96 ± 0.84) and late resistant (7.87 ± 1.48) cells of A2780 model. Combinatorial treatment of U0126 along with CisPac reduced LC3^+ve^LAMP1^+ve^ puncta by fivefold (5.3 ± 0.59 puncta/cell) in A2780Dual^ER^ cells (*n* = 35 cells/group). **F** Number of LC3^+ve^ puncta in sensitive, early and late resistant A2780 cells treated with CisPac (138.14 ± 11.98, 107 ± 6.95, 58.92 ± 4.96, respectively) alone or in combination U0126 (125.6 ± 10.79, 137.6 ± 10.62, 58.92 ± 5.65, respectively). **G**, **H** Graphical representation of surface area and volume of LC3 puncta in sensitive, early and late resistant A2780 cells treated with CisPac alone or in combination U0126. Increased surface area and volume of these puncta was observed in A2780Dual^ER^ cells post CisPac treatment (0.14 ± 0.003 and 0.06 ± 0.003 µm^3^, respectively) compared to untreated cells (0.06 ± 0.004 and 0.015 ± 0.002 µm^3^, respectively), which further enhanced upon application of U0126 and platinum–taxol (0.184 ± 0.007 and 0.083 ± 0.004 µm^3^, respectively). (*n* = 30–40 cells/group, average area and volume was calculated from ~5000 puncta, data represent mean ± SEM of at least two independent experiments, ns indicates nonsignificant, *p < 0.05, **p < 0.005, ***p < 0.0005 as calculated by unpaired *t* test). **I** Immunoblot depicting increased UVRAG level in A2780Dual^ER^ and OAW42Dual^ER^ cells treated with platinum–taxol compared to cells treated with U0126 and platinum–taxol. **J** Immunoblot depicting reduction in Rab7 level in A2780Dual^ER^ and OAW42Dual^ER^ cells treated with U0126 and platinum–taxol. **K** CisPac treatment reduced UVRAG and Rab7 in A2780Dual^ER/ERK1^ (shERK1) compared to parental counterpart.

In order to understand ERK1/2’s role in autophagosome–lysosome fusion, we utilized the classical mCherry-EGFP-LC3 reporter that shows yellow puncta [resulted due to colocalization of LC3 (green) and (mCherry)] after formation of autophagosomes and red puncta post autophagosome–lysosome fusion (as a consequence of quenching of GFP in the acidic environment of lysosome). A reduction in mcherry/EGFP ratio thus suggests reduction in autophagosome–lysosome fusion, while an increased ratio is indicative of higher autophagosome–lysosome fusion and increased autophagic flux^14^. CisPac treatment along with U0126 for 24 h in A2780Dual^ER^ cells stably expressing mCherry-EGFP-LC3 reporter reduced mcherry/EGFP ratio with an accumulation of yellow puncta in the perinuclear region, compared to only drug-treated cells, which predominantly displayed red puncta with fewer colocalization. Following similar treatment, A2780Dual^LR^ and sensitive cells showed significantly reduced red puncta and increased yellow puncta (Fig. 3A–C). Dual immunostaining with LC3 (autophagosome marker) and LAMP1 (lysosomal marker) revealed highest number of LC3^+ve^LAMP^+ve^ puncta in CisPac-treated A2780Dual^ER^ and OAW42Dual^ER^ cells compared to sensitive and Dual^LR^ cells, which reduced by 5- and 3.5-fold, respectively, after combinatorial treatment of U0126 and CisPac (Fig. 3D, E and Fig. S4A, B). CisPac treatment enhanced LC3^+ve^ puncta in A2780Dual^ER^ cells than untreated cells, which further increased after combinatorial treatment. Increased LC3^+ve^ puncta was also observed in A2780 cells post drug treatment, while least increase in number of LC3^+ve^ puncta was observed in Dual^LR^ post CisPac alone or combinatorial treatment (Fig. 3D, F). A similar trend was also observed in OAW42 model (Fig. S4A, C). Morphometric analysis of LC3^+ve^ puncta in chemoresistant models revealed an increased surface area and volume of these puncta in Dual^ER^ cells post CisPac treatment compared to untreated cells, which further enhanced upon application of U0126 and platinum–taxol. In contrast a marginal increase in surface area and volume of LC3^+ve^ puncta were observed in sensitive and Dual^LR^ cells of A2780 and OAW42 models post CisPac treatment alone or in combination with U0126 (Fig. 3D, G, H and Fig. S4A, D, E). UVRAG and Rubicon are involved in endocytic transport, autophagosome maturation, and/or autophagosome–lysosome fusion through Rab7 (refs. ^22–24^). Intriguingly, CisPac treatment enhanced both UVRAG and Rab7 level at 24 h which decreased post combinatorial treatment in both A2780Dual^ER^ and OAW42Dual^ER^ cells (Fig. 3I, J). The A2780Dual^ER/ERK1-KD^ cells also showed the similar trend in UVRAG and Rab7 (Fig. 3K). Similar treatments did not show any alteration in Rubicon level (Fig. S5). Inhibition of ERK1/2 or autophagy (CQ) increased PARP cleavage in A2780Dual^ER^ and OAW42Dual^ER^ cells (Fig. S6A–C). Real-time monitoring of cell death revealed a significantly higher and faster cell death kinetics post combinatorial treatment of CisPac and Trametinib in A2780Dual^ER^ and OAW42Dual^ER^ cells in comparison to only CisPac or only Trametinib treatment (Fig. S6D, E). An increase in both Annexin^+ve^ and Annexin^+ve^/PI^+ve^ cell population was observed post 12 and 24 h of CisPac + Trametinib treatment in comparison to only CisPac, only Trametinib-treated Dual^ER^ cells of both A2780 and OAW42 models (Fig. S6F–H).

### mtFL-p62 sensor portrays the role of ERK1/2 in p62 kinetics

**Fig. 4:**
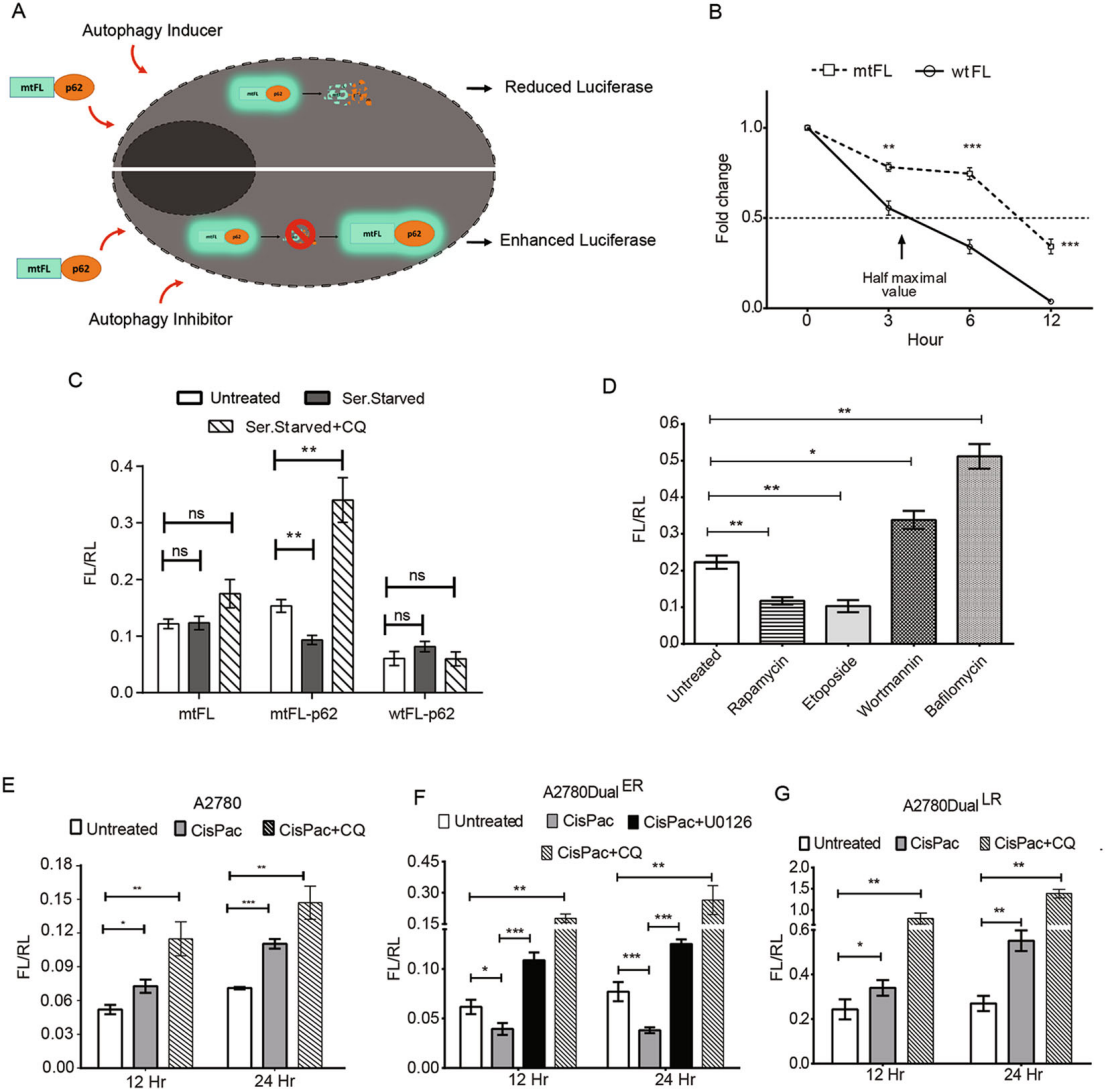
Tracking kinetics of stress-induced autophagy flux using mtFL-p62 construct. **A** Schematic representation of the working principle of mtFL-p62 as an autophagy sensor. **B** Luciferase activity in A2780 cells transfected with either mutated thermostable firefly luciferase (mtFL) or wild-type firefly luciferase (wtFL), and treated with cyclohexamide for 3, 6, and 12 h. Graph representing fold change in luciferase activity with respect to pretreatment condition (0 h) over time period. A 50% reduction in wtFL luciferase activity was observed post 3 h of cyclohexamide treatment, while mtFL showed similar reduction post 9 h. **C** Effect of serum starvation (Ser. Starved) alone or in presence of chloroquine (Ser. Starved + CQ) on luciferase activity in cells transfected with mtFL-p62, wtFL-p62, or only mtFL. Significant reduction (0.6-fold) in luciferase activity post 2 h of serum starvation and significant increase (2.2-fold) in luciferase activity post 2 h of serum starvation + CQ were observed in cells transfected with mtFL-p62, but not in cells transfected with either wtFL-p62 or only mtFL. **D** Graph representing reduced luciferase activity post Rapamycin and Etoposide (0.53- and 0.45-fold, respectively) treatment (24 h), while increased luciferase activity was observed upon Wortmannin and Bafilomycin (1.7- and 2.3-fold, respectively) treatment (24 h) in A2780 cells transfected mtFL-p62. **E**–**G** p62 degradation kinetics monitored by luciferase activity in A2780 chemoresistant model. CisPac treatment increase luciferase activity in A2780 (1.4- and 1.6-fold at 12 and 24 h) and A2780Dual^LR^ (1.3- and 2-fold at 12 and 24 h) cells, which further enhanced in presence of chloroquine (CisPac + CQ). Luciferase activity significantly reduced post 12 and 24 h of CisPac (0.6- and 0.5-fold) treatment, while CisPac + U0126 significantly increased luciferase activity by 2.76- and 3.29-fold in A2780Dual^ER^ cells.

The above results persuaded us to evaluate the role of ERK1/2 inhibitor in the regulation of drug-induced autophagic flux in vivo. Since the currently available sensors fail to monitor kinetics of autophagic flux in vivo in real time, we developed a novel luciferase-based autophagy sensor, mtFL-p62, as p62 degradation is well correlated with autophagic flux kinetics^25^. Increased p62 accumulation due to stalled autophagy would enhance luciferase activity, while p62 degradation due to increased autophagic flux would result in reduced luciferase activity (Fig. 4A). To avoid reporter protein-mediated p62 degradation, we utilized a mutant thermostable Firefly Luciferase (mtFL)^26^ which showed significantly longer half-life compared to wild-type firefly luciferase (wtFL; Fig. 4B). Serum starvation reduced mtFL-p62 reporter activity, while CQ treatment increased luciferase activity compared to the control. However, these modulations were not observed under similar conditions in cells transfected with only mtFL or wtFL-p62, implying the mtFL’s capacity to accurately portray autophagic flux through p62 kinetics (Fig. 4C). We further validated our sensor with known autophagy modulators like Rapamycin, which binds to FKBP12 and selectively inhibits mTORC1 to promote autophagy, Etoposides, which activates autophagic cell death in ATG5, Beclin1 and Bcl-xL-dependent manner, Wortmannin, a persistent class III PI3K inhibitor which inhibits induction of autophagy, and Bafilomycin, a potent lysosomal inhibitor which regulates lysosome acidification via vacuolar H^+^ATPase^15,27–30^. Rapamycin and Etoposide reduced luciferase activity, while Wortmannin and Bafilomycin increased luciferase activity in A2780 cells (Fig. 4D). Next mtFL-p62 was employed to monitor CisPac-induced autophagic flux in A2780 and OAW42 chemoresistant models. Platinum–taxol treatment enhanced luciferase activities in CisPac-treated A2780 and A2780Dual^LR^ cells, while a reduction in luciferase activity was observed in A2780Dual^ER^ cells when these cells were transiently transfected with the autophagy sensor. Combinatorial treatment of U0126 with platinum–taxol increased luciferase activity in A2780Dual^ER^ cells. Combination of CQ with CisPac for 12 and 24 h increased luciferase activity in all three cell lines (Fig. 4E–G). Similar trend was observed in the OAW42 chemoresistant cells (Fig. S7A–C). Further, proteasome inhibitor (Bortezomib) failed to rescue the CisPac-mediated reduction in mtFL-p62 luciferase activity in A2780Dual^ER^ cells, indicating specific role of autophagy in mtFL-p62 degradation (Fig. S7D).

### mtFL-p62 sensor tracks real-time autophagy kinetics in live cells and animals

**Fig. 5:**
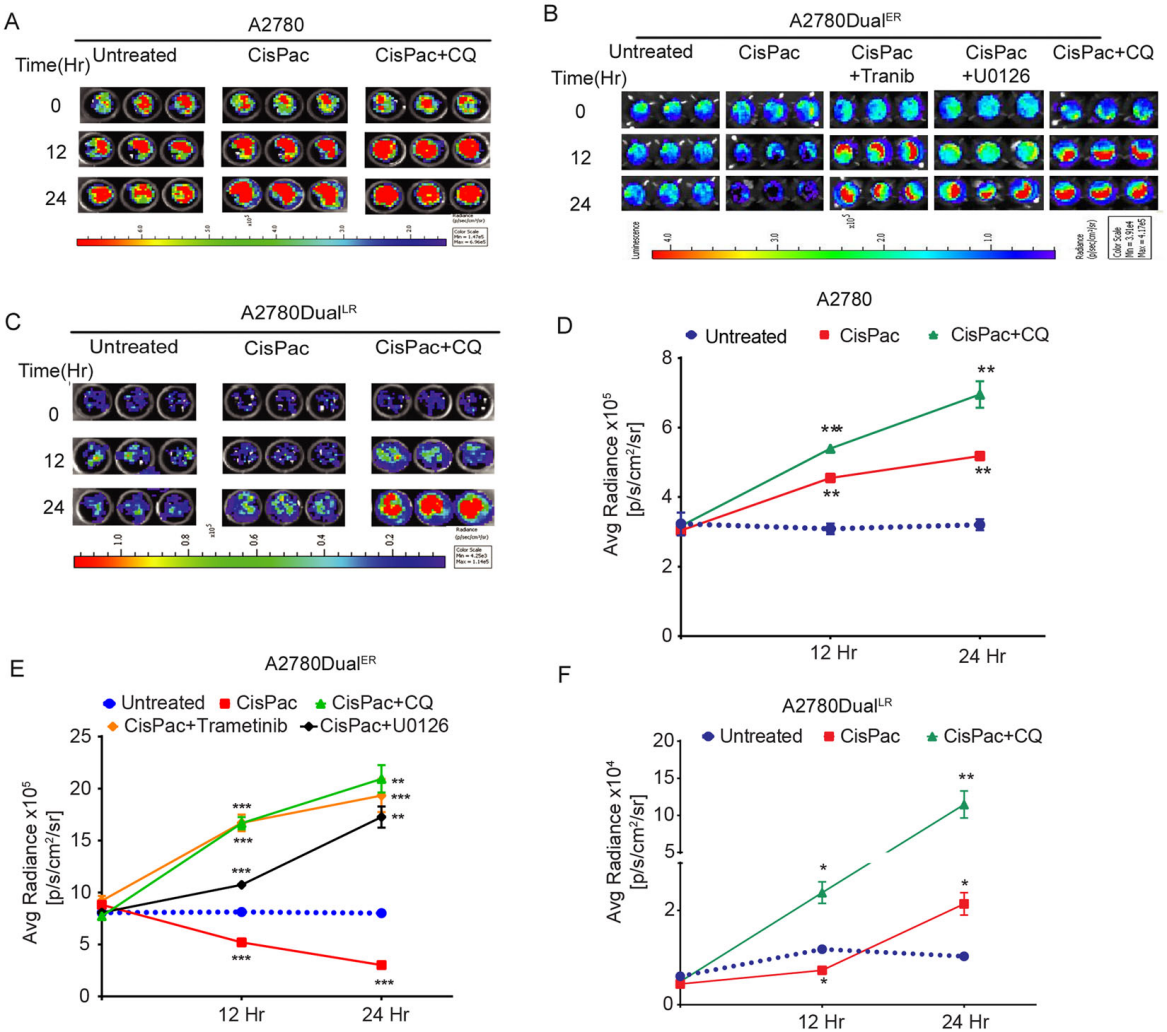
Live cell imaging of chemotherapy-induced autophagy flux utilizing mtFL-p62 construct. **A**–**C** Representative live cell images of autophagic flux in A2780, A2780Dual^ER^, and A2780Dual^LR^ cells expressing mtFL-p62 reporter post 12 and 24 h of CisPac treatment alone or with ERK1/2 inhibitor [U0126/Trametinib (Tranib)] or CQ. **D**–**F** Quantitation of luciferase signal from live cell imaging showed specific reduction (0.64- and 0.37-fold, respectively) in luciferase activity in A2780Dual^ER^ cells post 12 and 24 h of CisPac treatment, while combinatorial treatment of Trametinib and CisPac at similar time points increased luciferase activity (2.05- and 2.41-fold, respectively) in comparison to respective pretreatment condition (0 h). A similar increase in luciferase activity was also observed in cells treated with combination of U0126/CQ and CisPac. Platinum–taxol treatment for 12 and 24 h increased luciferase activity in A2780 (1.5- and 1.72-fold, respectively) and A2780Dual^LR^ (1.5- and 4.8-fold) cells, which further increased in presence of CQ in both the cell lines (data represent mean ± SEM of at least two independent experiments, ns indicates nonsignificant, *p < 0.05, **p < 0.005, ***p < 0.0005 as calculated by unpaired *t* test).

**Fig. 6:**
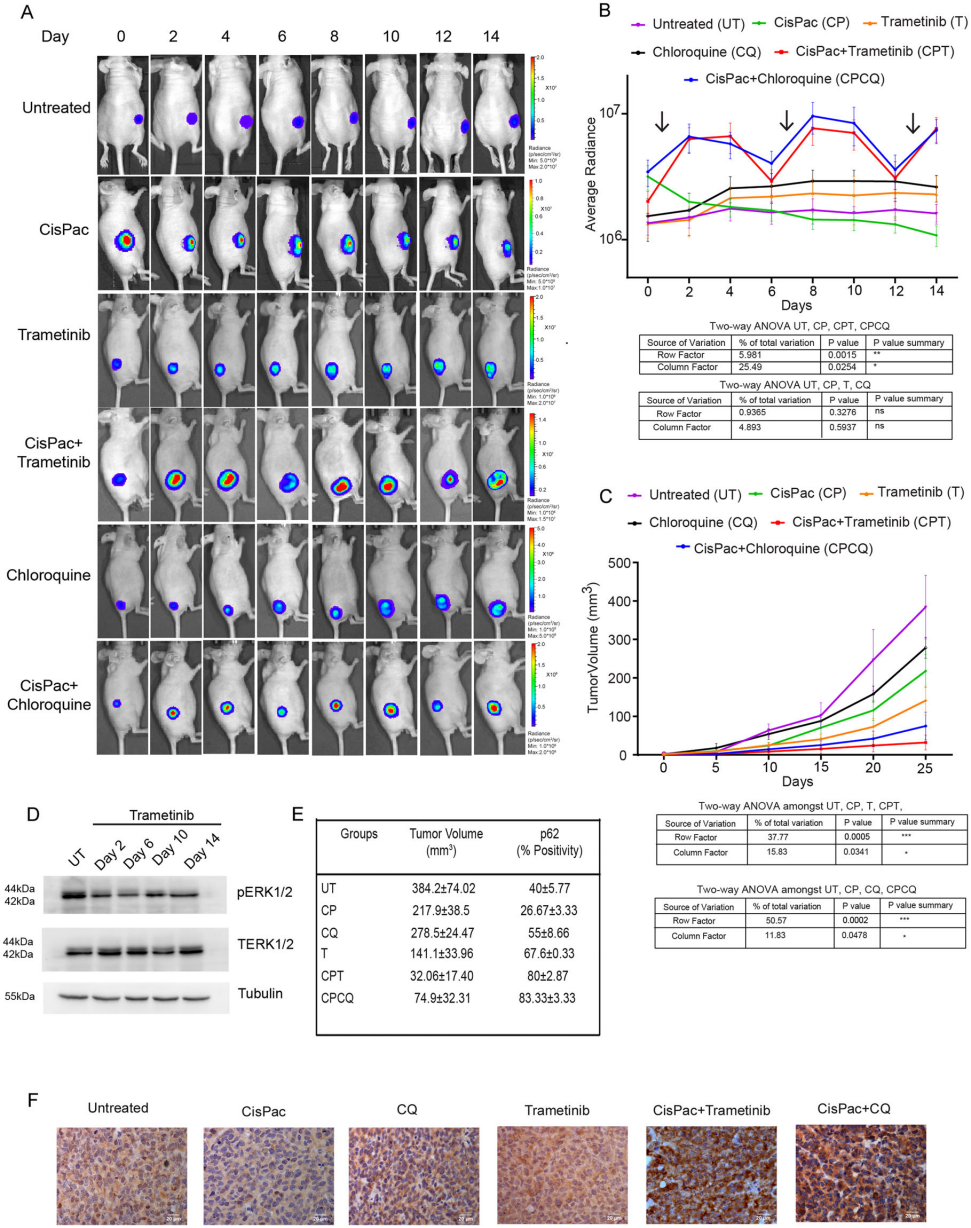
Noninvasive bioluminescence imaging of chemotherapy-induced autophagy flux in live animals. **A** Representative bioluminescence images of A2780Dual^ER^ tumor xenograft-bearing mouse expressing mtFL-p62 autophagy sensor treated with vehicle (untreated), CisPac, Trametinib, CQ, CisPac + Trametinib, and CisPac + CQ and monitored over 14 days. **B** Graphical representation of kinetics of quantified bioluminescence signal from mice (*n* = 8) showed Trametinib treatment alone enhanced (1.60-fold) bioluminescence on day 4 [(2.14 × 10^6^ ± 0.44 × 10^6^ vs. 1.33 × 10^6^ ± 0.28 × 10^6^ p/s/cm^2^/sr, (day 0)], which remained stationary till day 14 (2.29 × 10^6^ ± 0.85 × 10^6^ p/s/cm^2^/sr). A similar kinetics was observed with CQ treatment alone with a 1.66-fold increased luminescence on day 4 [(2.56 × 10^6^ ± 0.56 × 10^6^ p/s/cm^2^/sr vs. 1.54 × 10^6^ ± 0.53 × 10^6^ p/s/cm^2^/sr (day 0)] that remained stationary till day 14 (2.62 × 10^6^ ± 0.58 × 10^6^ p/s/cm^2^/sr). In contrary, CisPac treatment resulted in slow but continued reduction in bioluminescence [(3.17 × 10^6^ ± 0.65 × 10^6^ (day 0) to 2 × 10^6^ ± 0.31 × 10^6^ (day 2) to 1.82 × 10^6^ ± 0.28 × 10^6^ (day 4) to 1.08 × 10^6^ ± 0.18 × 10^6^ (day 14) p/s/cm^2^/sr), ~0.34-fold]. Application of Trametinib along with CisPac tripled luminescence signal by day 2 [(2.01 × 10^6^ ± 0.37 × 10^6^ (day 0) to 6.29 × 10^6^ ± 1.36 × 10^6^ (day 2)] then remained steady at day 4 (6.62 × 10^6^ ± 1.69 × 10^6^ p/s/cm^2^/sr) followed by a drop on day 6 (2.92 × 10^6^ ± 0.62 × 10^5^ p/s/cm^2^/sr), which again peaked [7.66 × 10^6^ ± 1.92 × 10^6^ (day 8) to 7.04 × 10^6^ ± 1.78 × 10^6^ (day 10) p/s/cm^2^/sr), ~3.49-fold] after second dose on day 7. The luminescence signal again increased at day 14 (7.62 × 10^6^ ± 1.59 × 10^6^) after third dose on day 12 (3.08 × 10^6^ ± 1.01 × 10^6^). Application of CQ along with CisPac also increased bioluminescence (~1.9-fold) till day 2 [(3.46 × 10^6^ ± 0.76 × 10^5^ (day 0) to 6.58 × 10^6^ ± 1.60 × 10^6^ (day 2)] which then gradually reduced till the administration of second combinatorial dose on day 7, following which a spike in bioluminescence was observed on day 8 (9.58 × 10^6^ ± 2.53 × 10^6^ p/s/cm^2^/sr, ~2.76-fold) and day 10 (8.41 × 10^6^ ± 2.6 × 10^6^ p/s/cm^2^/sr, ~2.43-fold). The luminesce signal again spiked on day 14 (7.38 × 10^6^ ± 1.49 × 10^6^ p/s/cm^2^/sr, ~2.13-fold; arrows indicate time points of CisPac intervention, ns indicates nonsignificant, *p < 0.05, **p < 0.005, ***p < 0.0005 as calculated by two-way ANOVA). **C** Graphical representation of alteration in tumor volume demonstrating combinatorial treatment of Trametinib/CQ along with CisPac show reduced tumor growth compared to untreated, only CisPac, only Trametinib, and only CQ group (ns indicates nonsignificant, *p < 0.05, **p < 0.005, ***p < 0.0005 as calculated by two-way ANOVA). **D** Immunoblot depicting downregulation of phospho ERK post daily administration of Trametinib treatment in mouse tumor on day 2, 6, 10, and 14. **E**, **F** Graphical representation of tumor volume and p62 percent-positive cells in mice tumor sections and immunohistochemical staining of p62 in mice tumor tissue with all the interventions mentioned above. Increased p62 and reduced tumor volume was observed in tissue section obtained from tumors treated with combination of CisPac along with Trametinib/CQ in comparison to tissue obtained from tumors treated with single agent. p62 stating intensity was lowest in tumor sections obtained from only CisPac group.

Real-time monitoring of chemotherapy-induced autophagic flux in live cells stably expressing the mtFL-p62 reporter also exhibited an enhanced luminescence signal in A2780 and A2780Dual^LR^ cells post 12 and 24 h, compared to pretreatment condition (Fig. 5A, C, D, F). U0126 is an old generation MEK1/2 inhibitor and which is not very effective in vivo, thus for further in vivo experiments we chose Trametinib over U0126 (ref. ^31^). A reduced luminescence signal was observed upon drug treatment in A2780Dual^ER^ cells which enhanced after combinatorial treatments of ERK1/2 inhibitor (U0126, Trametinib) and CisPac (Fig. 5B, E). CQ treatment along with CisPac increased signal output at all the stages (Fig. 5A–F). Next tumor xenografts of A2780Dual^ER^ cells stably expressing mtFL-p62 fusion reporter were developed in nude mice and therapy-induced autophagic flux was monitored for 14 days (Fig. 6A, B). Treatment of Trametinib alone showed 1.60-fold enhanced bioluminescence on day 4 over day 0, which remained stationary till day 14. A similar kinetics was observed with CQ treatment alone with a 1.66-fold increased luminescence on day 4 that remained stationary till day 14. On the contrary, CisPac treatment resulted in 0.62-fold reduction in luminescence signal on day 2, which again reduced further post second and third dose by 0.45- (day 8) and 0.32-fold (day 14), indicating ongoing p62 degradation and an active autophagic flux. When combinatorial treatment of Trametinib with CisPac was administrated, a 3.12-fold increase in luminescence signal was observed on day 2, which remain steady till day 4 and then dropped on day 6. Administration of second and third doses on day 7 and day 12, respectively, again induced a 3.80- and 3.78-fold increase in luminescence signal on day 8 and day 14, respectively, indicating the role of Trametinib in inhibition of CisPac-induced autophagic flux. Application of chloroquine along with CisPac also increased bioluminescence (1.9-fold) till day 2, which then gradually reduced till the administration of second and third combinatorial dose on day 7 and day 12, following which a 2.76- and 2.13-fold increase in bioluminescence was observed on day 8 and day 14, respectively. (Fig. 6A, B). Though demonstrating an incremental trend, the change of luciferase signals post treatment with single agents (CQ and Trametinib) over 14 days were not found to be statistically significant compared to control and CisPac-treated groups by two-way ANOVA analysis. This indicates that Trametinib and CQ may not be enough to block autophagy to a significant extent within 14 days. However, when the modulation in luminescence signals of the combinatorial groups (CisPac with Trametinib or CQ) were compared among days and with other groups by two-way ANOVA analysis, highly significant differences (*p* = 0.0015 and 0.02, respectively) were observed indicating such combinatorial treatments are sufficient to arrest autophagy in short duration. Trametinib treatment also reduced ERK1/2 activation as observed from tumor lysates by immunoblotting (Fig. 6D).

After administration of individual therapeutic regimen for 15 days, tumor growth was monitored till day 25. Single treatment of Trametinib showed 0.36-fold reduction in tumor volume while, only CisPac showed 0.56-fold reduction in tumor volume in comparison to untreated cells. Interestingly, combinatorial treatment of CisPac along with Trametinib significantly reduced (0.08-, 0.14-, and 0.22-fold at day 25) tumor volume in comparison to untreated, CisPac, and only Trametinib-treated group, respectively. Similar reduction in tumor volume was observed in CisPac + CQ group in comparison to untreated (0.19-fold), CisPac (0.34-fold), and only CQ (0.26-fold) group, indicating the effectivity of combinatorial treatment over single agent. Enhanced p62 staining was observed in tumors treated with Trametinib or CQ along with platinum–taxol, while a reduction in p62 staining was observed in CisPac-treated group (Fig. 6E, F).

## Discussion

Acquirement of resistance toward chemo/targeted/radiation therapy has become the most critical challenge to success of cancer therapy. Despite the presence of small subset of intrinsically resistant cells, majority of the cancer cells gradually acquire resistance through a series of molecular and biochemical alterations^21,32^. Understanding these gradual alterations may help in identifying a therapeutic window that can prevent development of a highly resistant disease. Utilizing the indigenously developed dynamic chemoresistant model of EOC^21^, we identified an active ERK1/2 signaling promotes platinum–taxol-induced autophagic flux at the onset of resistance, inhibition of which stalled autophagic flux by impairing autophagosome–lysosome fusion via UVRAG and Rab7 and increased cell death, apoptosis and chemo-sensitization. Autophagic flux diminishes at late/highly resistant stage due to the hyperactive AKT signaling. Further, we designed and validated a novel autophagy sensor (mtFL-p62), which indicated a chemotherapy-induced reduction in luciferase activity specifically in Dual^ER^ cells. Interestingly, ERK1/2 inhibition by two different inhibitors (U0126 for in vitro studies and Trametinib for live cell and live animal studies) with chemotherapy in Dual^ER^ cells simulated a comparable effect like chloroquine in both live cells and tumor xenograft.

Autophagy induction upon chemotherapeutic insult often leads to therapy resistance in majority of cancers including EOC^4,5,8,10,12^. Though multiple studies-associated cisplatin or paclitaxel resistance with enhanced autophagic flux mediated through multiple mechanisms like YAP oncoprotein, TXNDC17, and ERK1/2, none of the studies investigated the dynamic association of autophagy across the evolution of chemoresistance^12,33–35^. Here, we showed that both sensitive and Dual^ER^ cells exhibited proper initiation of autophagy as indicated by increased LC3II/LC3I ratio and increased the formation of phagophore and autophagosome, however, p62 degradation, which denotes the completion of autophagy, was only evident in the Dual^ER^ cells, which also showed increased number of autophagolysosomes post drug treatment. The Dual^LR^ cells showed reduced initiation and completion of autophagy presumably due to presence of hyperactivated AKT. Inhibition of AKT indeed promoted active autophagy in these cells as demonstrated by other studies^36–38^. TEM images also revealed lowest number of autophagic bodies in the Dual^LR^ cells. Our data indicate that an upregulated autophagic flux at the onset of resistance probably assists in dealing with initial chemotherapeutic stress and fosters development of a highly chemoresistant phenotype where autophagy becomes dispensable.

ERK1/2 activation is reported to regulate autophagy in contextual manner^7,9^. Chemotherapy-induced ERK1/2 activation positively regulates autophagic flux in multiple cancers, as well as in a panel of cisplatin-resistant ovarian cancer cells^10,35,39–41^. We observed chemotherapy-induced phospho-ERK1/2 in sensitive and Dual^LR^ cells but not in Dual^ER^ cells, which possessed highest basal level of activated ERK1/2. Pharmacological inhibition of ERK1/2 in Dual^ER^ cells increased LC3 and p62 accumulation indicating blockade at late stage of autophagy. Indeed, this blockade was confirmed by increased accumulation of autophagosomes with reduced autophagolysosomes in Dual^ER^ cells post CisPac + U0126 treatment. Increased LC3^+ve^LAMP1^+ve^ puncta and mCherry/EGFP-LC3 ratio are bonafide signatures indicating autophagolysosome formation^42,43^ were specifically observed in the Dual^ER^ cells post platinum–taxol treatment compared to sensitive and Dual^LR^ cells, which drastically reduced upon ERK1/2 inhibition, highlighting the role of ERK1/2 in ongoing autophagic flux and possibly in autophagosome–lysosome fusion. Blockade of autophagosome–lysosome fusion by vinblastine in amino acid starved CHO cell line increased autophagosome size^44^. Intriguingly, we also observed increased size (both area and volume) of LC3 puncta upon ERK1/2 inhibition in Dual^ER^ cells. Regulation of such phenotype by ERK1/2 is still not reported. Thus, our data indicate the role of basal ERK1/2 for maintenance of autophagy under therapeutic stress. ERK1/2 activation by lindane in sertoli cells was reported to prevent autophagosome maturation and targeted inhibition of Ras-Raf-MEK-ERK pathways in PDAC cells bearing activating mutation in *Ras* or *Raf*, or in BRAFi resistant brain tumor cells, was known to induce autophagic flux^45–47^. These apparent contradictions with our observation might have arose due to our model of chemotherapy-induced autophagy in chemoresistant cells compared to these studies, which used untransformed or cancer cells with hyperactivated Ras-Raf signaling. Cisplatin-induced ERK1/2 phosphorylation was reported to enhance autophagy in cisplatin-resistant A2780 cells (A2780/CP70)^12^. Though it is difficult to compare the level of resistance of A2780/CP70 with our resistant cells, ERK1/2 activation is associated with enhanced autophagic flux in both the studies, indicating that an optimal level of ERK activation (basal or drug induced) is critical for proper completion of autophagy. Inhibition of ERK activation reduced autophagic flux and promoted apoptosis and cell death.

A large number of proteins are involved in autophagosome maturation and autophagosome–lysosome fusion. Downregulation of UVRAG expression by pathogens like HCV or *Mycobacterium tuberculosis* or tissue-specific knockdown of UVRAG was reported to inhibit the autophagolysosome formation with subsequent autophagosome accumulation^48–50^. Interestingly, genetic or pharmacological inhibition of ERK1/2 clearly diminished UVRAG level upregulated by CisPac treatment in Dual^ER^ cells. Rab7 is a critical regulator of autophagolysosome fusion. Rab7 knockdown or overexpression of its dominant-negative mutant was reported to reduce LC3^+ve^LAMP1^+ve^ structure and led to the accumulation of large autophagosome in serum-starved Hela and CHO cells^51^. Increased size of autophagosomes upon combinatorial treatment of U0126 and platinum–taxol, and reduced Rab7 level by genetic or pharmacological inhibition of ERK1/2 indicated a mechanistic link between activated ERK1/2 and autophagosome–lysosome fusion in Dual^ER^ cells. Taken together our data suggest ERK1/2 activation regulates multiple components of autophagolysosome formation. A direct connection between ERK1/2 activation with autophagosome–lysosome fusion regulators has yet not been elucidated, but indirect connection through EGR1 or miR-138 holds the probable mechanisms^52^.

This stage-specific ERK1/2-mediated upregulation of autophagic flux during progression of platinum–taxol resistance clearly opens up a therapeutic window to combat chemoresistance. To evaluate efficacy of ERK1/2 inhibitors to block autophagy in preclinical mouse model, we developed a novel mtFL-p62 autophagy sensor. The available fluorescent-based sensors are only applicable either for live cells or ex vivo microscopic analysis of transgenic animals, but not for real-time monitoring in live animals^15,53,54^, while the only in vivo validated autophagy luciferase system utilized ratiometric quantitation of the degradation of aggregated polyQ80-luciferase to non-aggregated polyQ19-luciferase by electroporating the constructs in skeletal muscles of normal and ATG16L hypomorphic mice^20^. Such in vivo ratiometric analysis is not an easily adaptable system to monitor autophagy kinetics for drug discovery. The mtFL-p62 reporter does not require an additional reporter to measure the autophagy kinetics since the longer half-life of mtFL than wtFL potentially reduces the inaccuracy associated with autophagy-independent degradation of the reporter protein. Reduction in luciferase signal in serum-starved condition and in drug-treated Dual^ER^ cells and concomitant signal enhancement by combinatorial treatment (with either U0126 or CQ) signifies the power of this sensor in capturing the autophagy dynamics through p62.

The actual strength of this mtFL-p62 sensor lies in real-time monitoring of autophagic flux from live cells and preclinical mouse models, which has never been demonstrated by any of the existing autophagy sensors. In mice model, drug treatment gradually diminished the luciferase signal over 14 days signifying ongoing autophagic flux in the tumors, which was further reflected by immunohistochemical staining of p62 in tumor tissues. Compared to Trametinib or CQ treatment alone, which showed a nonsignificant increase in luciferase signal, combinatorial treatment of Trametinib or CQ with platinum–taxol resulted in more pronounced blockade in autophagic flux by 48–96 h. Intriguingly, Trametinib was found to block autophagic flux for a period of 24–96 h in a similar extent as CQ, a well-established autophagy inhibitor, indicating the potential of Trametinib in regulation of autophagic flux in clinical settings. Administration of CisPac along with Trametinib significantly reduced tumor volume and increased p62 staining intensity compared to untreated and single agent-treated group indicating the therapeutic potential of combinatorial regimen of platinum–taxol and Trametinib in management of platinum resistant ovarian cancer. Altogether our data suggest ERK1/2 inhibitors might be able to exert therapeutic benefit to EOC patients by regulating the autophagic flux in the tumor cells particularly after few cycles of platinum–taxol therapy, which is known to initiate resistance development. Our unique autophagy sensor would be a valuable resource for such clinical studies.

## Methods

### Cell lines and culture conditions

A2780 and OAW42 were cultured in DMEM (GIBCO, CA, USA, 12800017) and MEM, respectively (GIBCO, CA, USA, 61100061), supplemented with 10% fetal bovine serum (Himedia, INDIA, RM10409) and 1% pencillin–streptomycin (Himedia, INDIA, A002) in humidified atmosphere containing 5% CO_2_ at 37 °C. Sensitive, early and late resistant cells were treated with CisPac (Sigma-Aldrich, MO, USA, P4394/T1912) at a dosage ten times their respective IC50 (Table S1) for 12 and/or 24 h for all the experiments aimed at inducing high level of autophagy in short duration. U0126 (CST, CO, USA, 9910) or CQ (Sigma-Aldrich, MO, USA, C6628) treatments were performed at 10 µM concentration either alone or with CisPac. Trametinib (Cayman Chemicals, MI, USA, 16292) treatments were performed in vitro at 10 nM concentration. Cells were treated with Bortezomib (Sigma-Aldrich, MO, USA, 5.04314) at concentration of 1 µM for 12 and 24 h. Cyclohexamide (Sigma-Aldrich, MO, USA, 01810) treatment was done at concentration 10 µg/ml for 3, 6, and 12 h. Bafilomycin (Sigma-Aldrich, MO, USA, B1793) treatment was done at concentration 100 ng/ml, Wortmanin (Sigma-Aldrich, MO, USA, W1628) treatment was done at concentration 500 ng/ml, Rapamycin (Sigma-Aldrich, MO, USA, R0395) treatment was done at concentration 200 ng/ml, and Etoposide (Sigma-Aldrich, MO, USA, E1383) at concentration of 30 nM for 24 h.

### Development of chemoresistant model

Cisplatin–paclitaxel resistant A2780 and OAW42 cells were developed following pulse method strategy, with incremental doses of platinum–taxol over a period of 6 months^21^. Cells were treated with fixed concentration of CisPac for 2 h and then allowed to grow till 80–90% confluency in the drug-free medium. The survived fraction was then again challenged with same concentration of CisPac for three consecutive cycles followed by an incremental dose of three successive cycles. Cellular viability and development of resistance were determined at every stage till a highly resistant (~90% viable at IC_50_ of sensitive sells) phenotype was achieved. Based upon resistant index, cells were categorized into early resistant (Dual^ER^), i.e., cells at the onset of chemoresistance (five times higher than IC_50_ of sensitive cells) and late resistant (Dual^LR^), i.e., cells that achieved a highly resistant phenotype (ten times higher than IC_50_ of sensitive cells).

### Immunoblotting

A2780/OAW42 cells and their early and late resistant counterparts were treated with platinum and taxol alone or in combination with ERK1/2 inhibitor (U0126/Trametinib) or CQ for 12 and 24 h, as mentioned in results and figure legends. Cell lysates were prepared using RIPA buffer (Sigma-Aldrich, MO, USA, R0278) consisting of protease and phosphatase inhibitors (Sigma-Aldrich, MO, USA, PPC1010), followed by measurement of protein concentration by Bradford assay (Sigma-Aldrich, MO, USA, B6916). To determine the linear range of detection for LC3 conversion and p62 degradation, different amounts of proteins (15, 30, 45, and 60 µg for A2780 chemoresistant model and 15, 30, and 45 µg for OAW42 chemoresistant model) were loaded and separated on 8–12% polyacrylamide gel and transferred onto PVDF membrane (PALL, NY, USA, 741260). The membranes were blocked in 5% BSA in TBS with 0.05% Tween 20 (Sigma-Aldrich, MO, USA, P1379) for 1 h followed by incubation with primary antibodies overnight at 4 °C. Membranes were washed three times with TBST followed by incubation with HRP-conjugated anti-rabbit (Sigma-Aldrich, MO, USA, A6154) or anti-mouse secondary antibodies (Sigma-Aldrich, MO, USA, 4416) for 2 h at room temperature. The blots were developed using chemiluminescent substrate (Takara, CA, USA, T7101A) and images were captured in chemidoc imaging system (Biorad, CA, USA) and quantified using ImageJ software (NIH). A linear change in LC3 conversion, p62 degradation, and tubulin were observed with increasing amount of protein for both chemoresistant model (Fig. S8). A total of 30 µg of protein was found to be optimal to measure the LC3I–II conversion and p62 degradation for both A2780 and OAW42 chemoresistant models, and thus further used for the experiments. Primary antibodies: phospho/total ERK(4511p/ 4695 s), LC3B(2775 S), cleaved PARP(9541 S) were procured from CST (CO, USA), phospho and total p90^RSK1/2^ (AF892, AF2056) from R&D systems (MN, USA), Fra-1(sc-376148) from Santa cruz biotechnology (TX, USA), Rubicon (A9464) and UVRAG (A8462) antibodies from Abclonal (MA, USA), p62 from Abcam (UK, ab155686), phospho/total AKT (SAB5600064/SAB5600066) and tubulin(T5168) from Sigma-Aldrich (MO, USA) antibodies were used as per the manufacturer protocol.

### Transmission electron microscopy

Cell pellet were treated with 2.5% glutaraldehyde (volume:volume) in 0.1 M cacodylate buffer (pH: 7.4) for fixation, followed by postfixation for 1 h at 4 °C in 1% osmium tetroxide (weight:volume) in 0.1 M cacodylate buffer. Serial dehydration was performed through graded ethanol. Samples were embedded in araldite resin and polymerized at 70 °C for 24 h. The blocks were sectioned in ultrathin slices (50–70 nm) using Lieca UC7 ultramicrotome and mounted on 300 mesh copper grids. A 10% uranyl acetate alcoholic and lead citrate staining were performed for contrast enhancement. Samples were examined on a JEOL 1400plus transmission electron microscope operated at 120 kV. Images were acquired with iTEM software using Tengra camera. Based on the morphological and staining intensity the autophagic structures were quantified in 10–15 cells across different fields.

### Cell viability assay

A2780 and OAW42 chemoresistant cells were treated with respective IC50 dosage of CisPac either alone or along with U0126 for 48 h. Standard thiazolyl blue tetrazolium bromide (MTT, Sigma-Aldrich, MO, USA, M2128) method was used to calculate cell viability using the formula: Absorbance (Test)/absorbance (Control) × 100. Cells were incubated with 10% MTT solution for 2 h followed by addition of 100 µl DMSO and absorbance at 570 nm with background correction at 630 nm were measured^55^.

### Confocal and immunofluorescence microscopy

The mCherry-EGFP-LC3 (ptfLC3) plasmid was a gift from Tamotsu Yoshimori (Addgene, 21074; deposited by Yoshimori lab), which shows yellow puncta (due to colocalization of EGFP and mCherry) upon autophagosome formation, while red puncta (due to quenching of EGFP in lysosomal acidic pH) is observed upon formation of autophagolysosome, thus increased autophagic flux is characterized by reduced EGFP mCherry colocalization. While increase in autophagic flux increases mCherry/EGFP ratio due to reduction in EGFP puncta and increase in mCherry puncta. Stable clones of each stages of A2780 chemoresistant cellular model were developed by transfecting ptfLC3 plasmid with superfect reagent (Qiagen, DE, 301307) and selected against puromycin (Sigma-Aldrich, MO, USA, P8833). These clones were seeded over glass coverslips, treated with respective IC_50_ dosage of CisPac either alone or in combination with U0126 for 24 h and were fixed, using 4% paraformaldehyde (Sigma-Aldrich, MO, USA, 158127) for 10 min at 37 °C and mounted onto glass slides with Vectashield (Vector Labs, CA, USA, H-1000). Images were obtained using Carl Ziess LSM 780 confocal microscope using 488 and 633 laser, and were analyzed for colocalization of EGFP and mCherry signal using Zen image analysis software (Carl Ziess, DE). Extent of colocalization was quantified using the Pixel intensity spatial correlation analysis and the extent of colocalization was expressed as Manders colocalization coefficient. The values ranges between 0 to 1, where 1 represents perfect colocalization and 0 depicts no colocalization, and a value over 0.5 signifies the extent of proper colocalization. To calculate mCherry/EGFP ratio, number of individual EGFP and mCherry puncta were quantified using imageJ software. For immunofluorescent microscopy, cells were fixed with 4% paraformaldehyde after respective treatments and blocked using 3% BSA in PBS buffer for 30 min at room temperature. Cells were then incubated with LC3 (1:100 dilution) and LAMP1 (1:200 dilution, DSHB, IA, USA, H4A3) antibody overnight at 4 °C, washed three times with PBS followed by incubation in anti-rabbit dylight 488 (1:200, Invitrogen, CA, USA, 35552) and anti-mouse dylight 633 (1:200, Invitrogen, CA, USA, 35512) antibody for 2 h at room temperature. Cells were washed further with PBS and mounted on to glass slides using Vectashield. Images were obtained in Carl Ziess LSM 780 confocal microscope using 63× objective at 3× digital zoom using 488 and 633 laser. The numbers of LC3/LAMP1 puncta were calculated using Zen and ImageJ software. Measurement of LC3 puncta area was deduced from the pixel size using imageJ software and the diameter of each LC3 puncta was calculated using the formula: Area (*A* = 4πr^2^) with the assumption that each LC3 puncta are perfect sphere. The value of the diameter obtained above was used to calculate the volume using the formula: Volume (*V*) = 4/3πr^3^.

### ERK1 silencing by lentiviral-mediated sh-RNA constructs

ERK1 knockdown lentiviral cassette was developed using a target sequence (5′-GACCGGATGTTAACCTTTA-3′)^56^. Lentiviral particles were produced as described previously^57^. Briefly, lentiviruses carrying ERK1 target sequence were produced in 293FT cells by transfecting lentivector plasmid, P-delta packaging plasmid, and VSVG envelope plasmid in a ratio of 4:2:1 ratio. Post 60 h of transfection, viruses were collected, transduced in A2780Dual^ER^ cells, and cells stably expressing the shERK1 construct were enriched by FACS sorting by EGFP.

### Real-time analysis of cell death using CellTox^TM^

Real-time cytotoxicity of platinum–taxol, Trametinib, and their combination was evaluated by CellTOX Green® cytotoxicity assay (Promega, WI, USA, G8741). Breifly, A2780Dual^ER^ and OAW42Dual^ER^ (5000 cells/well) cells were plated overnight in black clear bottom 96-well plate. Cells were then treated as mentioned in the results section along with CellTox green dye (1:1000) for 54 h. Dead cell fluorescence was measured every 6 h at 485–500 nm_Ex_/520–530 nm_Em_ in Biotek cytation5 (VT, USA) imaging system.

### Apoptosis detection with Annexin/PI

For detection of apoptosis, Dual^ER^ cells of both A2780 (1 × 10^5^) and OAW42 (0.5 × 10^6^) model were stained with Annexin V-conjugated FITC and propidium iodide using BD FITC Annexin V apoptosis detection kit (BD Pharmingen^TM^ CA, USA, 556547), as per manufactures protocol. Briefly, cells were harvested in ice-cold PBS and then resuspended in 100 µl of Annexin V binding buffer, followed by addition of 5 µl of Annexin V-FITC and 5 µl of PI. After 15 min of incubation in dark at room temperature, 400 µl of Annexin V binding buffer was added to each tube and analyzed (25–50,000 events) in Attune NxT Flow cytometer (ThermoFisher MA, USA). Flow cytometry data was analyzed using FlowJo version 10 software.

### Plasmid construction

GFP-p62 plasmid was purchased from Addgene (#38277, developed by Dr. Noboru Mizushima and deposited by Mizushima Lab). The p62 open reading frame was first cloned into pcDNA3.1+ vector at EcoR1 restriction site followed by insertion of mtFL gene at NheI and HindIII restriction sites upstream of p62 to generate pcDNA3.1-CMV-mtFL-p62 construct. Next mtFL was replaced by FL2 to generate pcDNA3.1-CMV-FL-p62 fusion reporter.

### Luciferase assay

To measure the stability of mtFL and firefly luciferase (FL), pCMV-mtFL and pCMV-FL plasmids were transiently transfected in A2780^DualER^ cells followed by cyclohexamide treatment for 3, 6, and 12 h. The cells were lysed using passive lysis buffer (Promega, WI, USA, E1941) and luciferase activity was measured using luciferase assay system (Promega, WI, USA, E4030) in Biotek cytation5 imaging system (VT, USA). To measure the p62 degradation kinetics in A2780 and OAW42 chemoresistant model were transiently transfected with mtFL-p62 construct and pCMV-Renila luciferase construct (9:1), and luciferase readout was calculated as a ratio of FL/renilla luciferase (RL) using luciferase assay system and coelenterazine as measured in Biotek cytation5 imaging system^58^. All transfection experiments were performed in triplicates and repeated thrice.

### Live cell imaging

For live cell imaging, 5000 cells expressing mtFL-p62 were seeded into 96-well black plates, treated with CisPac alone or in combination with ERK1/2 inhibitor (U0126/Trametinib) or CQ for 12 and 24 h. Live cell imaging was performed as described earlier, using D-luciferine (1 mg/ml, Biosynth, MA, USA, L8220) in IVIS spectrum imaging system (Perkin-Elmer, MA, USA). Bioluminescence signals were quantified using the Live Image (4.4) software^58^.

### In vivo imaging

Animal care and euthanasia were performed as per Institutional Animal Ethics Committee approval of ACTREC. A total of 3 × 10^6^ A2780Dual^ER^ cells stably expressing mtFL-p62 construct, generated using G418 (Sigma-Aldrich, MO, USA) selection, were injected subcutaneously in female CD1-NUDE mice (6–8 weeks old, weight 20–24 grams) and divided into six following groups (*n* = 8) for monitoring p62 dynamics over 14 days upon treatment with single and combinatorial agents, group I: untreated mice, group II: CisPacl (animals treated with 2 mg/kg cisplatin + 1 mg/kg paclitaxel thrice on day 1, day 7, and day 12), group III: Trametinib (animals treated with 1 mg/kg of Trametinib daily for 15 days), group IV: CQ (animals treated with 40 mg/kg of CQ daily for 15 days), group V: Trametinib + CisPac (animals were treated with 1 mg/kg of Trametinib daily for 15 days and CisPac were administrated on day 1, day 7, and day 12), and group VI: CisPac + CQ (animals treated with 40 mg/kg of CQ daily for 15 days and with CisPac on day 1, day 7, and day 12). All the drugs were injected intraperitoneally. Animals were injected with D-luciferin (30 mg/kg) intraperitoneally and imaged alternate days in Xenogen-IVIS imaging systems. Data analysis and further image processing were carried out using Living Image software 4.4.

### Immunohistochemistry

IHC was performed as mentioned earlier^59^. Briefly, 5-µm thick sections of tumors were deparaffinised, hydrated, and treated with peroxide (Abcam, UK ab236466), which was followed by heat induced epitope retrieval in sodium isocitrate buffer (pH 8 for p62). For p62, antigen retrieval was carried at 120 °C for 6 min in a pressure cooker. The sections were then incubated with the protein blocking reagent (Abcam, UK) and treated with anti-p62 (Abcam, UK; 1:100; 4 °C for overnight). The sections were further treated with the secondary antibodies and developed using HRP-conjugated DAB substrate (Abcam, UK). Grading was done based on the intensity and the extent of positivity as scored by an experienced pathologist.

### Statistical analysis

Each experiment was repeated at least three times and data has been represented as mean ± SEM. Students *t* test was applied to evaluate the significance of the changes observed. For in vivo experiments, two-way ANOVA was applied to determine statistically significant changes across the groups. A *p* value < 0.05 was considered statistically significant.

## Supplementary information

Supplemental Materials
