# Supplementary material for: Molecular imaging of the kinetics of hyperactivated ERK1/2-mediated autophagy during acquirement of chemoresistance
Source: Cell Death Dis. 2021 Feb 8;12(2):161. doi: 10.1038/s41419-021-03451-y (PMC7870816; doi:10.1038/s41419-021-03451-y)
Supplement: Supplementary file 1 — Supplemental Materials [file 41419_2021_3451_MOESM1_ESM.pdf]

Supplementary Figures

Figure S1

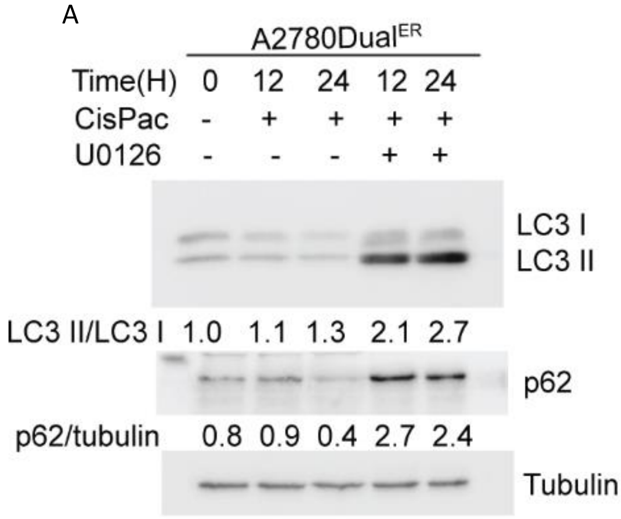

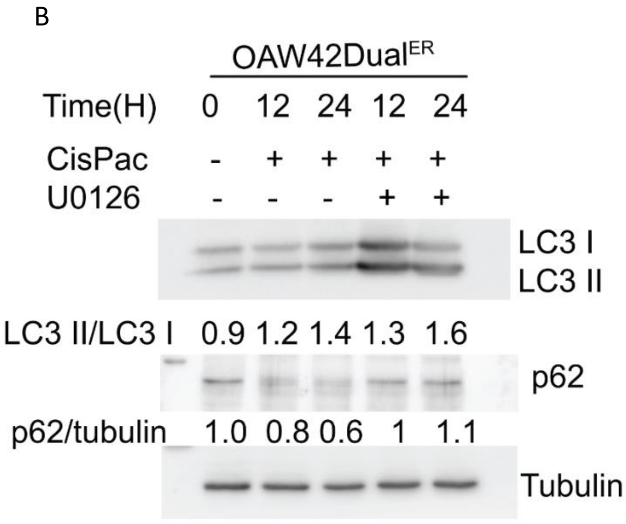

FigureS1A-B: ERK1/2 inhibition inhibits platinum-taxol induced autophagic flux:

Immunoblot depicting increased LC3 I-II conversion and p62 accumulation in A2780Dual^{ER} and OAW42Dual^{ER} cells treated with U0126 and platinum-taxol (CisPac) for 12 and 24 hours.

Figure S2

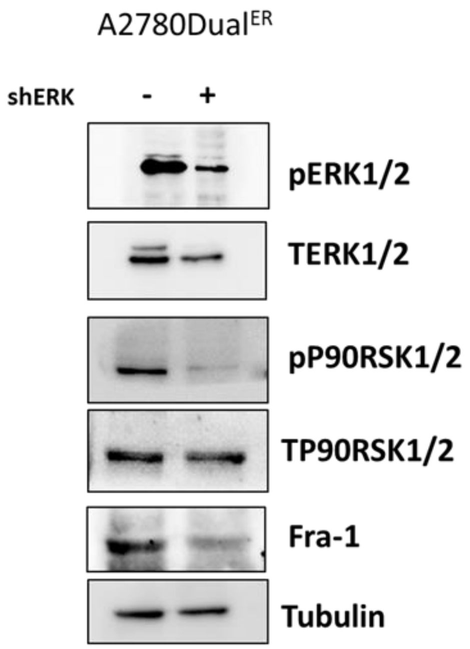

FigureS2: Immunoblot depicting efficiency of ERK1 knockdown in A2780Dual^{ER} cells expressing shERK1 lentiviral construct

Figure S3:

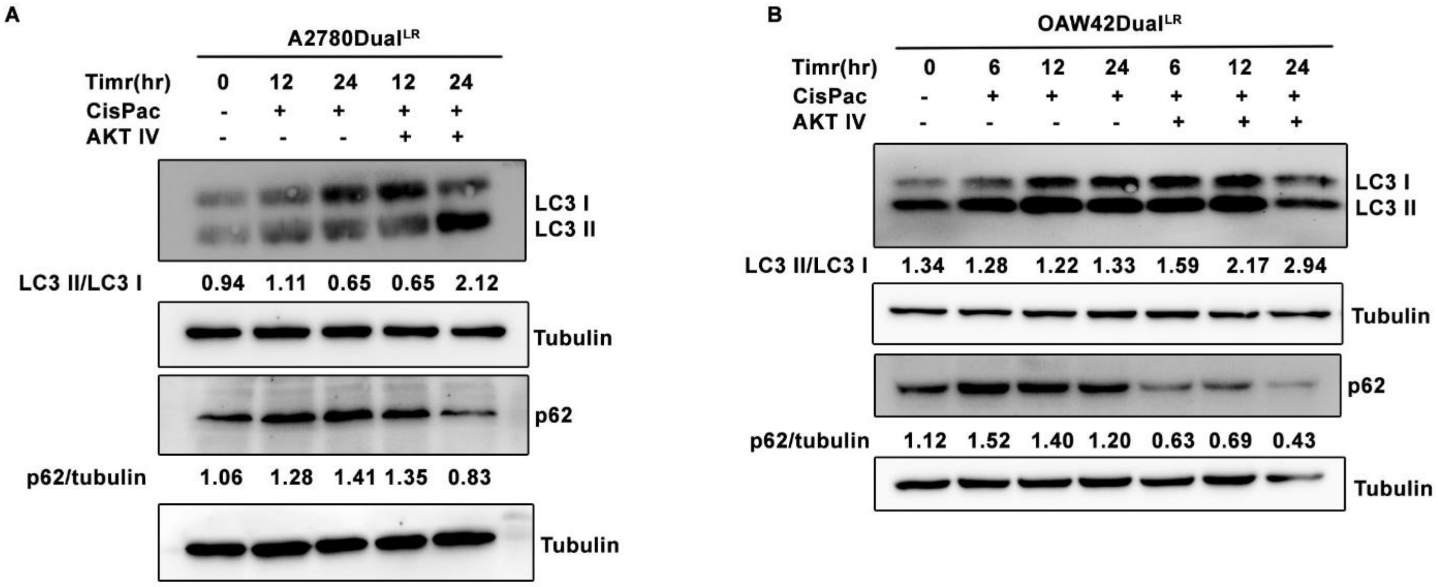

Figure S3: AKT inhibition positive regulates autophagic flux

(A, B) Immunoblot depicting increased LC3 I-II conversion and p62 degradation in A2780Dual^{LR} and OAW42Dual^{LR} cells upon combination treatment of AKT inhibitor (AKT IV, 200nM) and cisplatin-paclitaxel at different time points

Figure S4

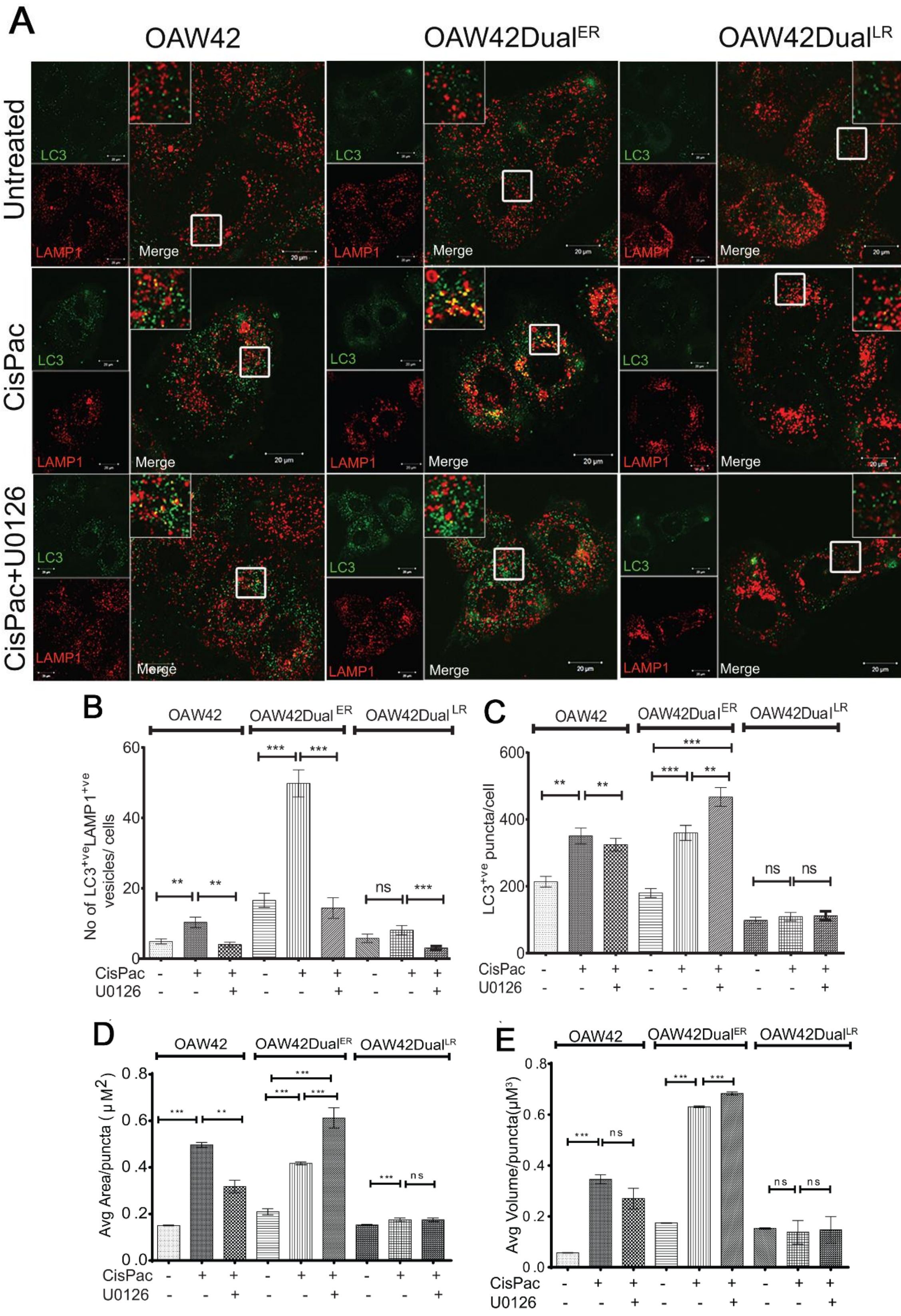

Continued.

FigureS4: ERK1/2 inhibition prevents autophagosome-lysosome fusion

A) Dual immunostaining of LC3 (Green, autophagosome) and LAMP1 (Red, lysosome) in sensitive, early and late resistant OAW42 cells exhibit differential autophagolysosome (yellow) numbers (B, C) Graphical representation of the number of LC3^{+ve}LAMP1^{+ve} puncta and LC3^{+ve} in sensitive, early and late resistant OAW42 cells treated with CisPac alone or in combination U0126. (G, K) Graphical representation of surface area and volume of LC3^{+ve} puncta in sensitive, early and late resistant OAW42 cells treated with CisPac alone or in combination U0126 (n=30-40 cells/group, average area and volume was calculated from ~ 5000 puncta, data represents mean ± SEM of at least two independent experiments, ns indicates non-significant,* indicates $p < 0.05$, ** $p < 0.005$, ***$p < 0.0005$ as calculated by unpaired t-test)

FigureS5

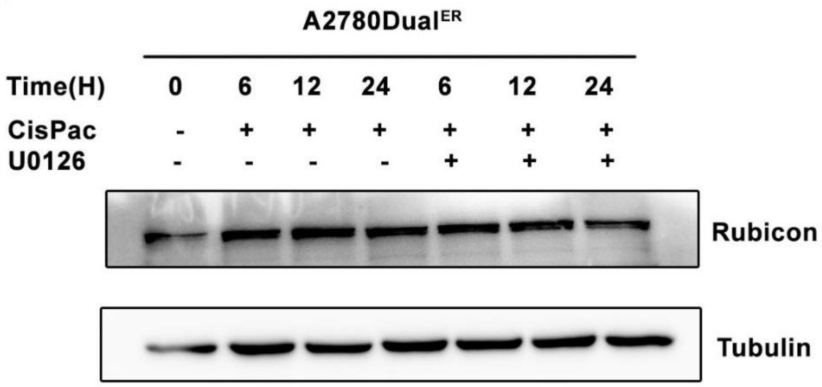

Figure S5: Immunoblot depicting no alternation in Rubicon level in A2780Dual^{ER} cells treated with cisplatin-paclitaxel alone or in combination of U0126

Fig S6:

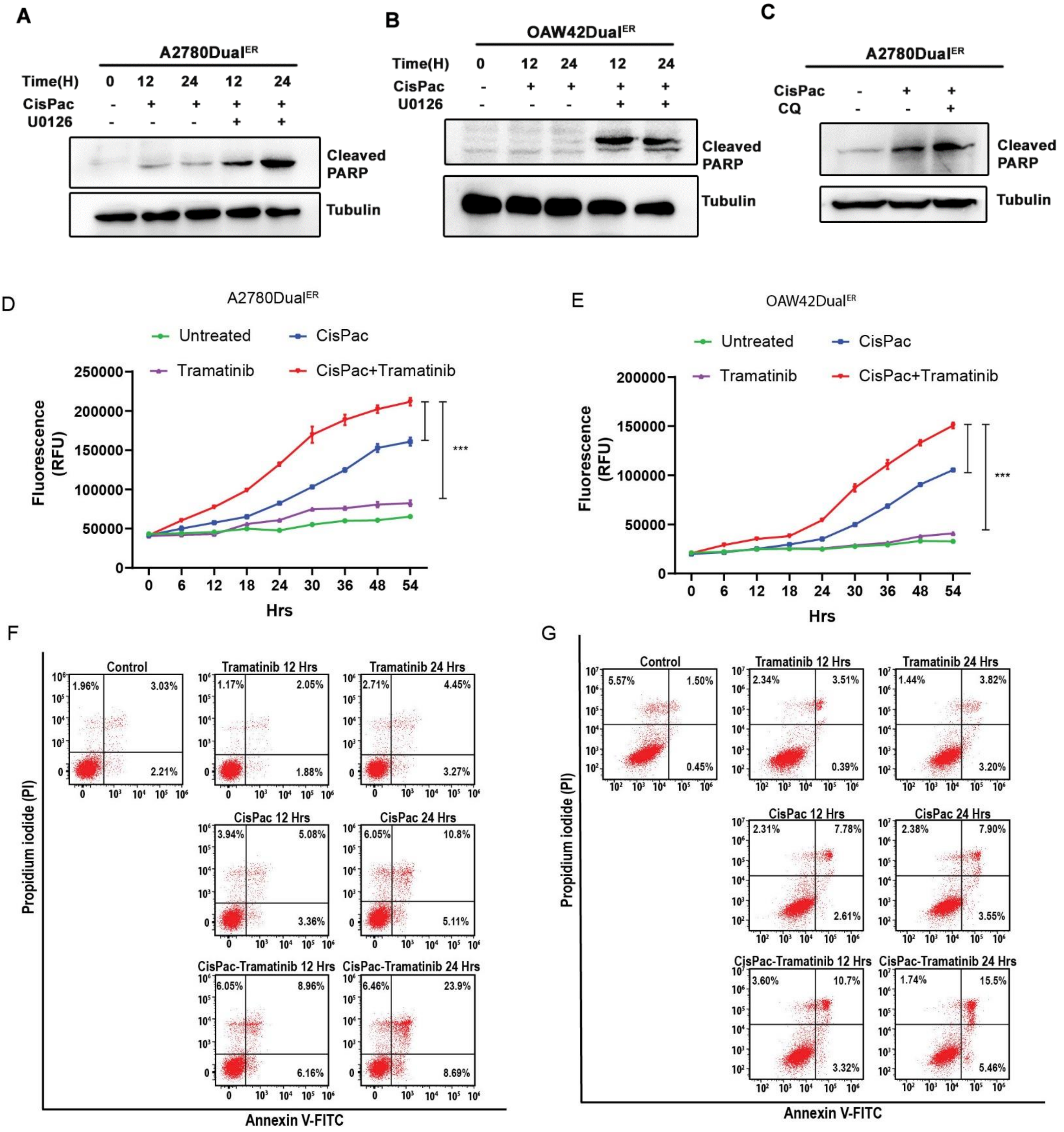

Continued.

H

A2780Dual^{ER}			
Cell line and Time	Early Apoptosis (EA)	Late Apoptosis (LA)	Total Apoptosis (EA+LA)
Untreated	2.21	3.03	5.24
CisPac 12 Hr	3.36	5.08	8.44
CisPac 24 Hr	5.11	10.8	15.91
Trametinib 12 Hr	1.88	2.05	3.93
Trametinib 24 Hr	3.27	4.45	7.72
CisPac+Trametib 12 Hr	6.16	8.96	15.12
CisPac+Trametinib 24 Hr	8.69	23.9	32.59
OAW42Dual^{ER}			
Untreated	0.45	1.5	1.95
CisPac 12 Hr	2.61	7.78	10.39
CisPac 24 Hr	3.65	7.9	11.55
Trametinib 12 Hr	0.39	3.51	3.9
Trametinib 24 Hr	3.2	3.82	7.02
CisPac+Trametib 12 Hr	3.32	10.7	14.02
CisPac+Trametinib 24 Hr	5.46	15.5	20.96

FigureS6: ERK1/2 inhibition sensitize Dual^{ER} cells to platinum-taxol induced apoptosis **(A-C)** Enhanced PARP cleavage in A2780Dual^{ER} and OAW42Dual^{ER} cells treated with platinum-taxol and U0126/chloroquine (CQ) for 12 and/or 24 hours. **(D-E)** Real-time cell death kinetics indicating significantly increased cell death post combinatorial treatment of CisPac+Trametinib in comparison to only platinum-taxol (CisPac), only Trametinib and untreated in A2780Dual^{ER} and OAW42Dual^{ER} cells. **(F-G)** Scatter plot showing time dependent increase in Annexin^{+ve} (early apoptotic) and Annexin^{+ve}/PI^{+ve} (late apoptotic) cells post combinatorial treatment of CisPac+Trametinib in comparison to only CisPac, only Trametinib and untreated cells in A2780Dual^{ER} and OAW42Dual^{ER} cells. **(H)** Table representing percentage of Annexin^{+ve} (early apoptotic) and Annexin^{+ve}/PI^{+ve} (late apoptotic) cells post combinatorial and single treatment.

Figure S7:

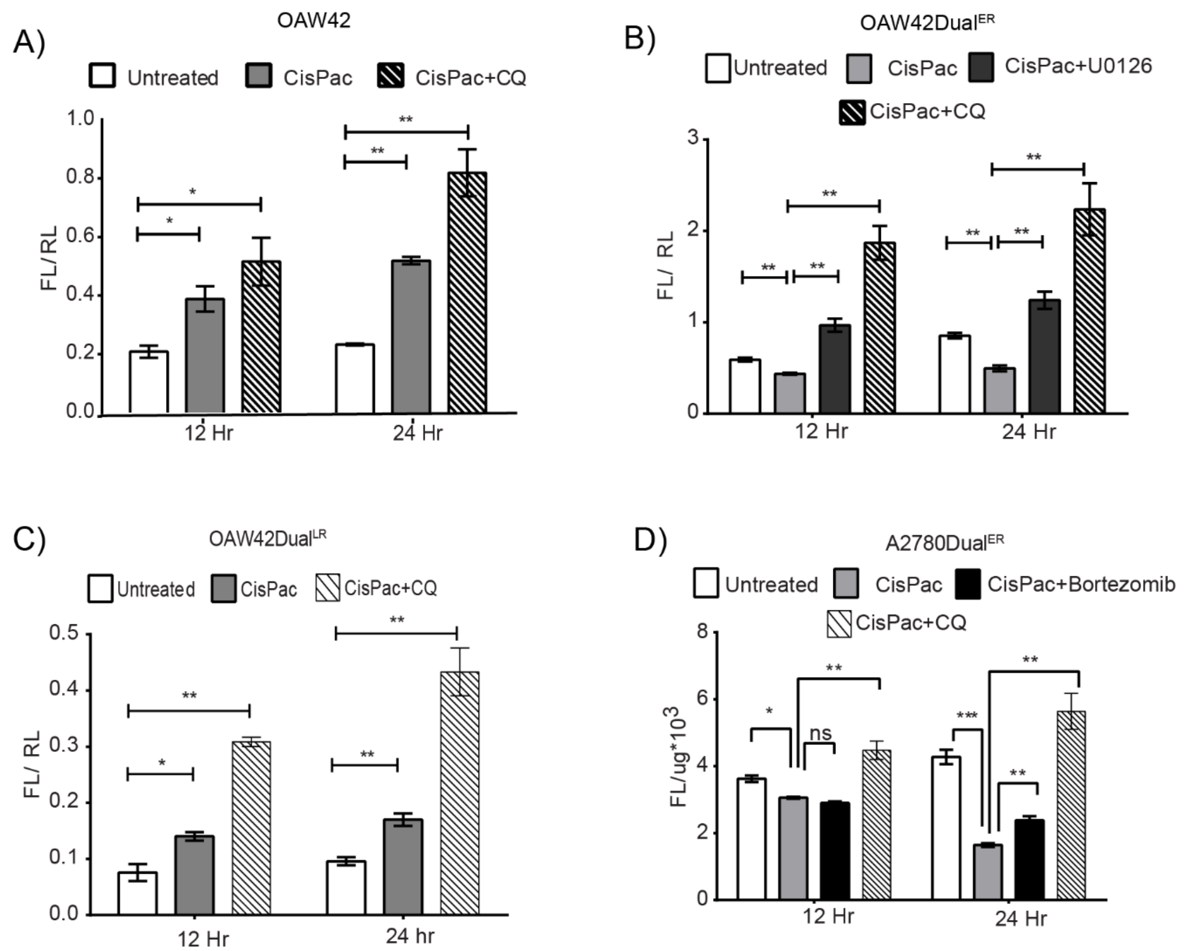

FigureS7:

(A) p62 degradation kinetics monitored by luciferase activity in sensitive OAW42 cells show a significantly increased luciferase activity 1.9- and 2.1-fold post 12 and 24 hours of CisPac treatment respectively which further enhanced in presence of chloroquine (CisPac+CQ). **(B)** Luciferase activity in OAW42Dual^{ER} cells expressing mtFL-p62 reporter increased by 2.2 and 2.5 fold post 12 and 24 hours of CisPac+U0126 treatment in comparison to cells treated with only CisPac. **(C)** CisPac treatment significantly increased luciferase activity in OAW42Dual^{LR} cells by 1.9 and 1.8 fold post 12 and 24 hours of treatment, further increase in luciferase activity

was observed upon combinatorial treatment of CisPac+CQ. **(D)** Proteasome inhibition for 12 and 24 hours along with CisPac (CisPac+Bortezomib) failed to rescue only CisPac mediated reduction in luciferase activity while application of chloroquine along with CisPac increased luciferase activity at both 12 and 24 hours. (Data represents mean ± SEM of at least two independent experiments, ns indicates non-significant, * indicates $p < 0.05$, ** $p < 0.005$, ***$p < 0.0005$ as calculated by unpaired t-test)

Figure S8

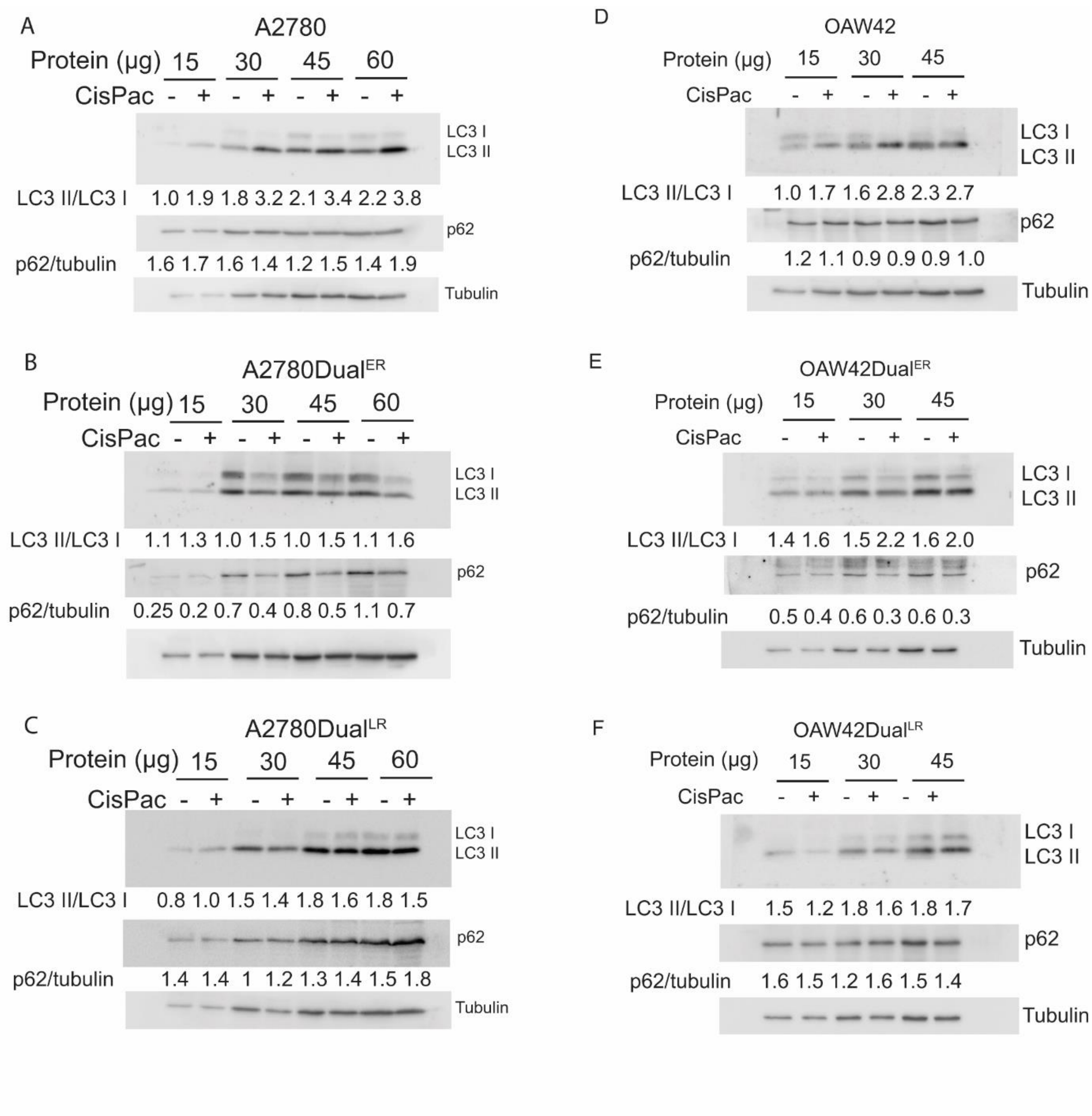

Figure S8: Linear range of LC3, p62 and tubulin detection in A2780 and OAW42 chemoresistant model

(A, B, C) Immunoblot depicting linear increase in band intensity of LC3, p62 and Tubulin with increasing amount of protein loaded (15, 30, 45 and 60 µg) for A2780 and its chemoresistant counterparts in both untreated and cisplatin-paclitaxel treated conditions. For further experimentation, 30 µg of protein was used as LC3, p62 and tubulin were optimally detected

at this amount in all the three stages of A2780 chemoresistant cells (D, E, F) Similarly, a linear increase in band intensity of LC3, p62 and Tubulin was observed with increasing amount of protein loaded (15, 30 and 45 μg) for OAW42 and its chemoresistant counterparts in both untreated and cisplatin-paclitaxel treated conditions. 30 μg of protein was used for LC3, p62 and tubulin for further experimentation as these proteins were optimally detected at this amount in all the three cell lines of OAW42 chemoresistant cells

Table S1:

Cell line	IC_{50} of Cisplatin-Paclitaxel
A2780	50ng/ml Cisplatin+ 8.5ng/ml Paclitaxel
A2780Dual^{ER}	250ng/ml Cisplatin+ 42.5ng/ml Paclitaxel
A2780Dual^{LR}	500ng/ml Cisplatin+ 85ng/ml Paclitaxel
OAW42	72ng/ml Cisplatin+ 14ng/ml Paclitaxel
OAW42Dual^{ER}	360ng/ml Cisplatin+ 70ng/ml Paclitaxel
OAW42Dual^{LR}	720ng/ml Cisplatin+140ng/ml Paclitaxel

Table S1: Tabulation of IC_{50} of induvial cell lines of A2780 and OAW42 cisplatin-paclitaxel dual resistant model.
